# A database of common vampire bat reports

**DOI:** 10.1038/s41597-022-01140-9

**Published:** 2022-02-16

**Authors:** Paige Van de Vuurst, M. Mónica Díaz, Annia Rodríguez-San Pedro, Juan Luis Allendes, Natalie Brown, Juan David Gutiérrez, Heliot Zarza, Stefan V. de Oliveira, Elsa Cárdenas-Canales, Rubén M. Barquez, Luis E. Escobar

**Affiliations:** 1 Department of Fish and Wildlife Conservation, Virginia Tech, Blacksburg, VA USA; 2 Consejo Nacional de Investigaciones Científicas y Técnicas (CONICET), Instituto de Investigaciones de Biodiversidad Argentina (PIDBA), Facultad de Ciencias Naturales e Instituto Miguel Lillo, Universidad Nacional de Tucumán, Tucumán, Argentina; 3 Centro de Investigación e Innovación Para el Cambio Climático (CiiCC), Facultad de Ciencias, Universidad Santo Tomás, Santiago, Chile; 4 Programa Para La Conservación de Murciélagos de Chile (PCMCh), Santiago, Chile; 5 Virginia-Maryland College of Veterinary Medicine, Virginia Tech, Blacksburg, VA USA; 6 Universidad de Santander, Facultad de Ingeniería, Grupo Ambiental de Investigación Aplicada-GAIA, Bucaramanga, Colombia; 7 Departamento de Ciencias Ambientales, CBS, Universidad Autónoma Metropolitana Unidad Lerma, Lerma de Villada, Mexico; 8 Department of Collective Health, Federal University of Uberlândia, Urberlândia, Minas Gerais Brazil; 9 Department of Pathobiological Sciences, School of Veterinary Medicine, University of Wisconsin-Madison, Madison, USA; 10 Instituto de Investigaciones de Biodiversidad Argentina (PIDBA), Facultad de Ciencias Naturales, Universidad Nacional de Tucumán, Tucumán, Argentina; 11 Global Change Center, Virginia Tech, Blacksburg, VA USA; 12 Center for Emerging Zoonotic and Arthropod-borne Pathogens, Virginia Tech, Blacksburg, VA USA

**Keywords:** Biogeography, Viral infection

## Abstract

The common vampire bat (*Desmodus rotundus*) is a sanguivorous (i.e., blood-eating) bat species distributed in the Americas from northern Mexico southwards to central Chile and Argentina. *Desmodus rotundus* is one of only three mammal species known to feed exclusively on blood, mainly from domestic mammals, although large wildlife and occasionally humans can also serve as a food source. Blood feeding makes *D. rotundus* an effective transmissor of pathogens to its prey. Consequently, this species is a common target of culling efforts by various individuals and organizations. Nevertheless, little is known about the historical distribution of *D. rotundus*. Detailed occurrence data are critical for the accurate assessment of past and current distributions of *D. rotundus* as part of ecological, biogeographical, and epidemiological research. This article presents a dataset of *D. rotundus* historical occurrence reports, including >39,000 locality reports across the Americas to facilitate the development of spatiotemporal studies of the species. Data are available at 10.6084/m9.figshare.15025296.

## Abstract (summary)

**Table:** 

Measurement(s)	occurrence report
Technology Type(s)	digital curation
Sample Characteristic - Organism	Desmodus rotundus
Sample Characteristic - Location	North America • South America

Machine-accessible metadata file describing the reported data: 10.6084/m9.figshare.18745316

## Background & Summary

The common vampire bat, *Desmodus rotundus* (É. Geoffroy, 1810) is a member of the family Phyllostomidae, subfamily Desmodontinae^1^. *Desmodus rotundus* is endemic to the Neotropics, where it occurs from northern Mexico, through all of Central America, and across most of South America^2,3^. *Desmodus* *rotundus* is found over an elevational range from sea level up to 3600 m in the Andes mountains^4^. Two subspecies are recognized: *D. r. rotundus* (Trinidad and Tobago, Colombia, Venezuela, the Guianas, Ecuador, Peru, Brazil, Bolivia, Paraguay, Argentina, Uruguay, and central Chile) and *D. r. murinus* (Mexico, Central America, northern and western Colombia, and western Andean slopes in Ecuador and Peru)^2^. *Desmodus rotundus* is a strictly sanguivorous species that feeds mainly on the blood of medium to large-bodied terrestrial mammals and some birds^5^. While groups such as humans and cattle are not natural prey of *D. rotundus*, they have been documented as being fed upon by this species^6,7^. The most common prey species of *D. rotundus* are peccaries, deer, tapirs, horses, cattle, pigs, and goats, and to a lesser extent, species such as chickens, dogs, and sea lions^4,8–10^.

*Desmodus rotundus* uses various landscapes throughout its broader geographic distribution, including open grasslands, savannas, tropical, subtropical, and dry forests, and even desert environments^2,8,11–15^. *Desmodus rotundus* can also adapt to different landcover types, and has been found not only in patches of old-growth or undisturbed forests, but also in disturbed areas such as agroforestry plots, silvopastoral systems, pastures, and secondary forests^13,16,17^. *Desmodus rotundus* usually roosts in small groups, from as few as 10 to a few hundred individuals, but can also be found roosting in groups of up to a few thousand individuals^2^. *Desmodus rotundus* also uses a variety of roosts, including tree holes, crevices, caves, and abandoned mines and houses^2^. The conservation status of *D. rotundus* was defined as of “Least Concern” in 2015 by the International Union for Conservation of Nature and Natural Resources (IUCN) Red List of Threatened Species, as it is presumed to be a common species with large and stable populations^18^.

*Desmodus rotundus* can act as a natural reservoir for various microorganisms with zoonotic potential, such as bacteria, including *Bartonella* spp.^19^, coronaviruses^20,21^, and rabies virus^12,22^. *Bartonella* spp. bacteria are globally distributed and have been known to cause endocarditis in humans and other animals^19,23,24^. Endocarditis is an infection of the inner lining of the heart, and can potentially be lethal^23,24^. Furthermore, several variations of coronaviruses have been identified in *D. rotundus*^20,25^. In the Americas, bats are considered to be a key reservoir of the rabies virus^26,27^, with *D. rotundus* being the main species responsible for transmitting rabies to livestock^28^. It has been estimated that bovine rabies transmitted by vampire bats causes the death of thousands of cattle annually, resulting in economic losses of hundreds of millions of dollars in Latin America^29,30^. Indirect costs associated with *D. rotundus* related rabies include the vaccination of millions of cattle as a preventative measure, and post-exposure treatments (rabies immunoglobulin serums and vaccination) for people exposed to *D. rotundus* bites^29,30^. The perpetuation of rabies in livestock may also be associated with the abundance and distribution of *D. rotundus*^28,31,32^, as sex-related (male) dispersal may contribute to the expansion of rabies virus into new areas^33^. Thus, the addition of livestock to the landscape promotes suitable conditions for *D. rotundus* breeding and feeding^28,34–36^.

Due to its reservoir status for potential pathogens*, D. rotundus* is considered to be a major public health problem in the tropical and subtropical regions of the Americas. Public health concerns are particularly prevalent in Amazonian regions, where many people live in vulnerable housing^37,38^ and human diseases transmitted by *D. rotundus* remain high^39^. In fact, since 2020, rabies has been included in the World Health Organization 2021–2030 road map as a zoonotic disease, and now requires coordination of mitigation strategies at the regional, national, and global levels^40,41^. Numerous outbreaks in rural human communities have been reported in Amazonian regions, including Peru^42^, Brazil^6,43^, and French Guiana^7,44^.

Several Latin American countries have developed programs to reduce the number of *D. rotundus* bites to humans and livestock^45^. Culling campaigns to reduce *D. rotundus* populations; however, have not proven useful in reducing the seroprevalence of rabies within vampire bat colonies^46^. It has been suggested that *D. rotundus* geographic distributional expansion is linked to landscape heterogeneity, degradation, and agricultural aggregations^13,37,47^. Nevertheless, an increase of suitable areas under future climatic scenarios may contribute to the increased risk of rabies in some regions of the Americas^34^. The study and analysis of *D. rotundus* occurrence data are, therefore, critical for the development of preventive measures for vampire-transmitted rabies^48,49^. This article presents a comprehensive dataset of curated historical occurrence reports of *D. rotundus* across the Americas to facilitate spatiotemporal modeling and other relevant *D. rotundus* research. The dataset is available at 10.6084/m9.figshare.15025296 [Ref. ^50^].

## Methods

**Fig. 1:**
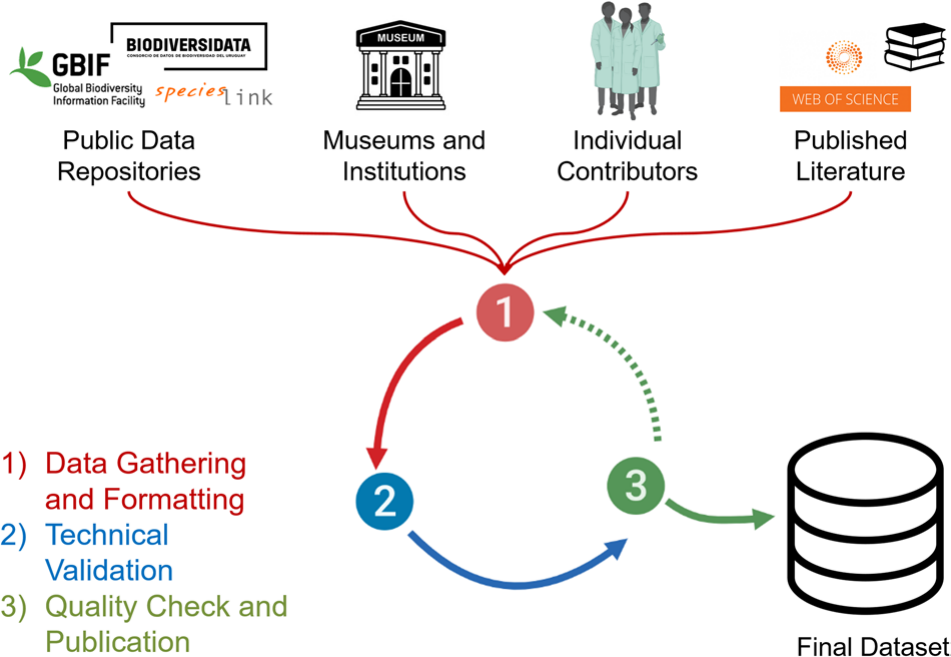
Dataset Workflow. Visual representation of the data curation, formatting, technical validation, and quality control process of this dataset of *Desmodus rotundus* occurrence reports. 1) Red: the data were first collected from various sources, including public data repositories, natural history museums and institutions, individual contributors, and published scientific literature. These data were formatted to meet Darwin Core archive standards. 2) Blue: the formatted data were then examined for error and underwent the technical validation process where reports which did not meet inclusion criteria were removed (see Technical Validation section). 3) Green: the data were manually examined again after the technical validation process for quality and to detect any remaining errors. This process was repeated as new *D. rotundus* occurrence reports were collected (dashed arrow). The final dataset was published at the end of this repeated validation and quality check process and is accessible from: 10.6084/m9.figshare.15025296.

**Fig. 2:**
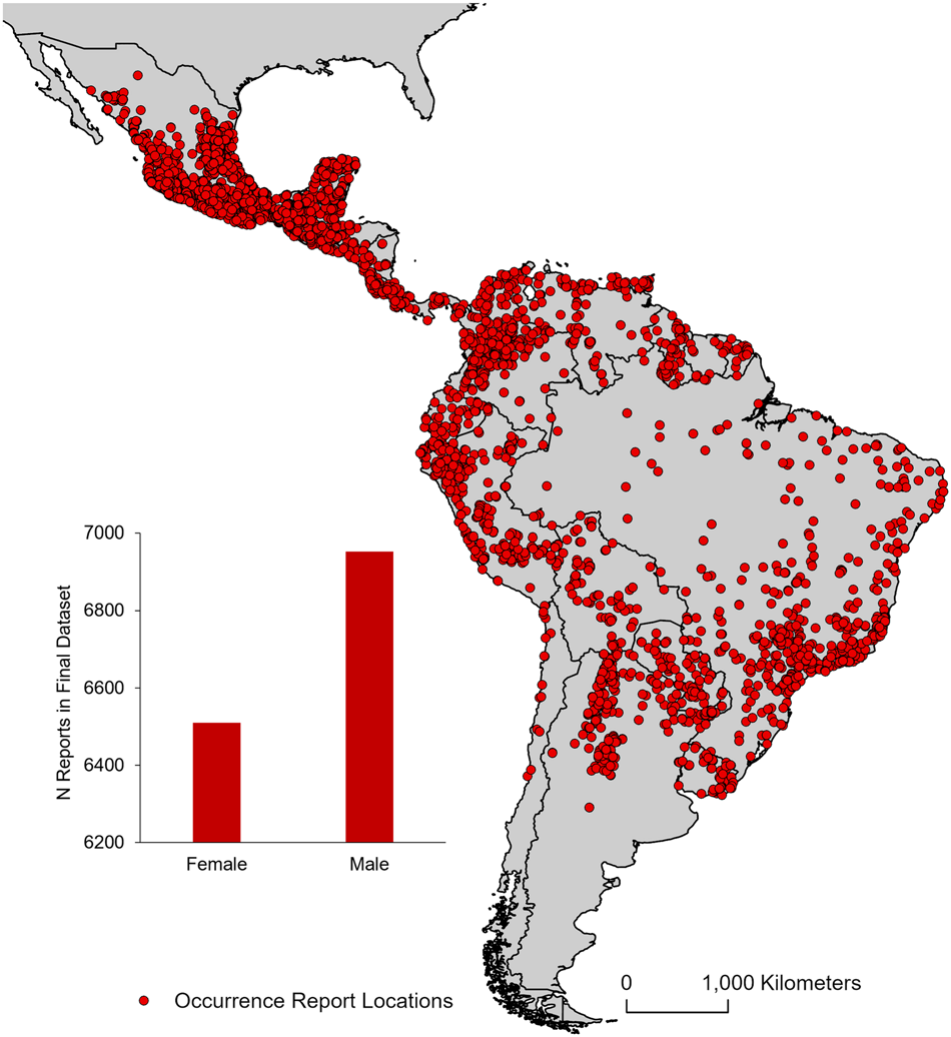
Map of occurrence report locations. Geographic locations of all occurrence reports in the final *Desmodus rotundus* dataset (red points) representing 7576 unique geographic locations available from the 39120 original reports. Inset: number of reports by sex in the final dataset (red bars) showing more reports of male individuals than females.

Data gathering for this dataset began in January 2020 and ended in December 2021. Occurrence reports of *D. rotundus* were collected from a variety of publicly available resources and databases, from a network of natural history museums across North, Central, and South America, from official repositories in ministries of agriculture and health, from published scientific literature across Latin America, and from privately held databases from individual contributors (Fig. 1). The final dataset includes 39120 individual occurrence reports (i.e., recorded instances where one or more *D. rotundus* individuals were recorded or observed) (Fig. 2) and 7576 unique geographic locations of *D. rotundus* existence. All data were collected in Darwin Core Archive format^51^. The Darwin Core Archive is a biodiversity and taxonomy based data definition format that makes use of standardized terms and file structures^51^. The use of the Darwin Core Archive allows for better data accessibility and mobilizations, as well as facilitates the data’s compliance with intercommunity standars^51,52^. *Desmodus rotundus* occurrence reports were geo-referenced using the World Geodetic System 1984 coordinate system in decimal degree units. Inclusion criteria for this dataset were:

A) That the report consisted of the modern species *Desmodus rotundus*^1^.
B) The report consisted of at least one individual.
C) The report had a recorded geographic coordinate (e.g., latitude and longitude), or a detailed locality description from which the occurrence could be geolocated (i.e., at finer detail beyond municipality level).
D) The report was from a validatable database, museum record, published piece of literature, machine recording (e.g., acoustic monitor or camera), human observation, preserved specimen, or live specimen.

**Fig. 3:**
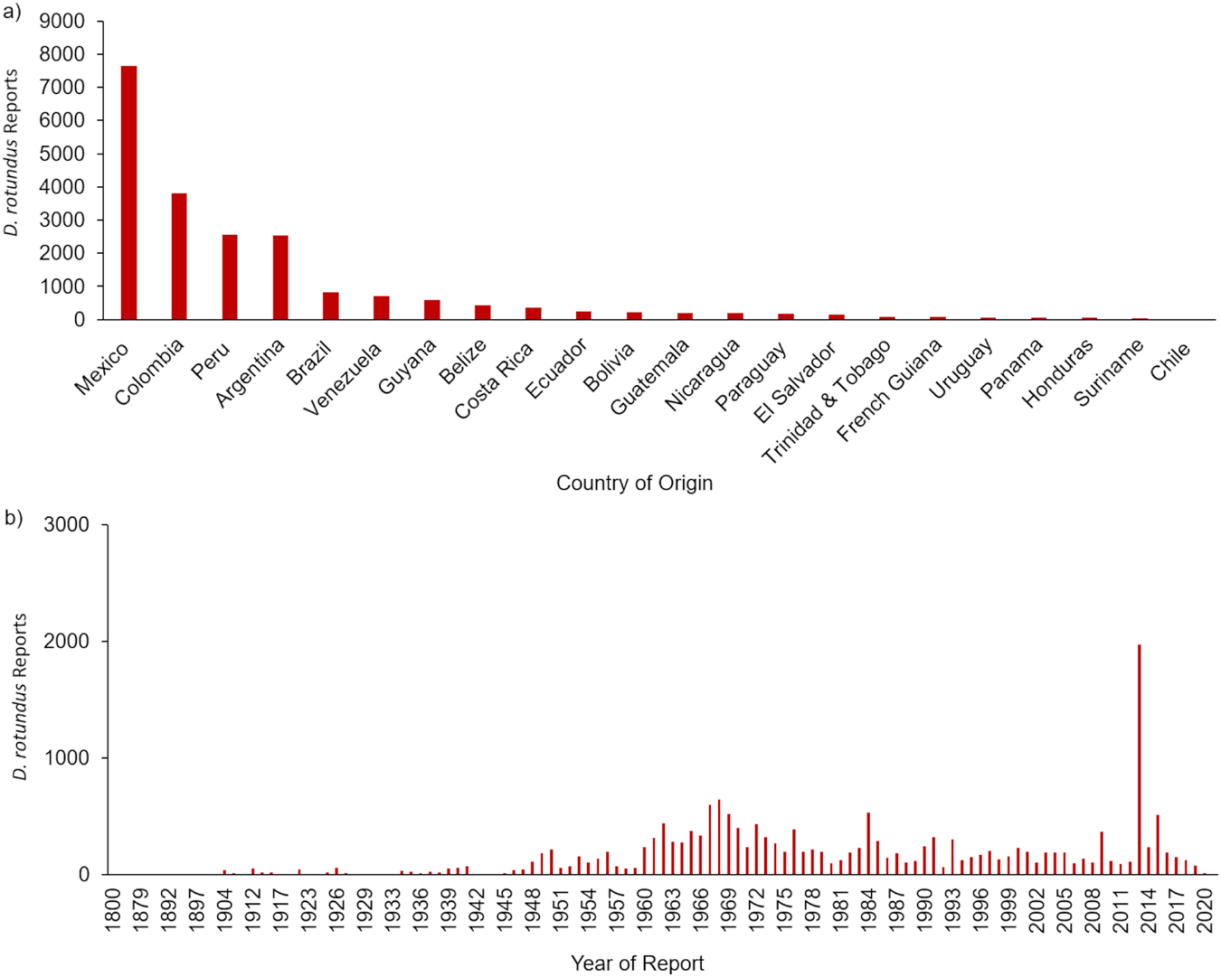
Data distribution by country and year. (**a**) The number of occurrence reports in the final *Desmodus rotundus* dataset is summarized based on the country in which the report occurred. The country with the most recorded occurrences was Mexico (n = 7653), followed by Colombia, Peru, and Argentina, which contributed over 2000 *D. rotundus* occurrence reports each. (**b**) Number of occurrence reports in the final *D . rotundus* dataset shown based on the year in which *D. rotundus* were recorded.

Metadata such as individual count (i.e., number of individuals recorded at each occurrence location, which may vary based on how the original report was collected), specimen age or life stage, basis of record, and date of capture were collected for each report whenever possible (Fig. 3). For occurrence reports where full metadata were not available or unable to be confirmed, the information was left blank in the final file (Supplementary Materials). Definitions for the database and metadata can be found in Online-only Table 1. After data gathering and technical validation the dataset was published in the Figshare data repository for public access (available at: 10.6084/m9.figshare.15025296)^50^.

## Data Records

To collect occurrence reports from published literature, a review was conducted of all publications available in the Web of Science literary repository on August 28, 2020 (Clarivate^TM^, 2020. available from: https://apps.webofknowledge.com/Search). We conducted a keyword search of topics in journal manuscripts, proceedings papers, and official reports. Keywords included “*Desmodus rotundus*”, “vampire bat”, and “common vampire bat”, and resulted in 315 manuscripts. The resulting manuscripts in Spanish, English, and Portuguese were then screened for associated *D. rotundus* occurrence data. A summary of these literature data sources can be found in Online-only Table 2. Additional reports were obtained from 37 institutions or researchers with privately held data. These contributors are summarized in Online-only Table 3. Data curation and validation followed the standardized protocol used for other data sources (see Technical Validation).

Occurrence reports were also collected from publicly available data repositories or databanks (i.e., web-based sources which centrally house data from other sources)^53^. These repositories included the Global Biodiversity Information Facility (GBIF)^54^, Biodiversidata^55^, and speciesLink^56^. Occurrence reports for *D. rotundus* were downloaded from the GBIF on October 30, 2020^54^. GBIF occurrence reports with coordinates which were located in the western hemisphere (n = 12865) were downloaded from the database in Darwin Core Archive format^51^. Occurrence reports based on fossil specimens (n = 3) were removed. After the cleaning and validation process (see Technical Validation), the final number of occurrences from GBIF was 12736. Originating datasets which contributed to the GBIF download are summarized in the Supplementary Materials. Occurrence reports from Biodiversidata (Uruguayan Consortium of Biodiversity Data Repository)^55^ were downloaded in December of 2020 from the vertebrate mammal sub dataset (n = 67)^55^. Occurrence reports from speciesLink (Centro de Referência em Informação Ambiental) were downloaded in August of 2021 (n = 2578). Of these reports, 918 were found to be already present in the GBIF database. In total, 1660 occurrences from speciesLink were added to the final dataset. A total of 298 occurrence reports without recorded coordinates were also downloaded from the publicly available data repositories and 48 of these reports were able to be georeferenced based on their locality descriptions. The other 250 could not be located due to a lack of detail in their locality descriptions and were therefore excluded. Georeferencing was completed using the *tidygeocoder* package in R^57^. All data are stored in the finalized dataset in the Figshare data repository for public access^50^.

## Technical Validation

To validate the collected data, we identified “redundant” reports (i.e., unique reports present in more than one dataset repository). Occurrence reports were flagged as redundant when the occurrence geolocation information (i.e., latitude, longitude, locality, and elevation) and institutional information (e.g., institutional identification number, originating dataset, institutional code, etc.) were identical. Other metadata such as date of occurrence, individual count, sex, life stage, and basis of record were used to confirm or reject redundancy. Reports where these variables matched were flagged and manually investigated to confirm redundancy. When redundant reports were found and confirmed, the original source occurrence report was retained. This process was completed using the *dyplr* package and base functions such as duplicate and unique in R^58,59^.

All occurrence reports were also investigated to eliminate occurrences with errors in geolocation using the *coordinateCleaner* package^60^. Using the functions cc_cap, cc_cen, cc_gbif, and cc_inst, we identified and removed occurrence reports which were erroneously assigned to country capitals, country centroids, or the GBIF/Biological Institution headquarters^60^. We also used cc_zero and cc_val functions to identify and remove reports with 0 latitude and 0 longitude as the geographic coordinates, or other invalid geographic coordinates (i.e., non-numeric or not possible coordinates such as Northern latitudes over 90°)^60^. The remaining occurrences were then visualized in geographic space. Occurrence reports which were located outside of the American continents (i.e., in the ocean) were identified and flagged. These occurrences were then investigated manually for errors. Coordinates that were identified as suspicious spatial outliers (>500 km from their nearest neighbor) were validated by contacting the publishing institution or individual (e.g., the natural history museum, collection, or author). Mapping was done using the package *ggplot2* in R and ArcGIS Pro software^59,61,62^.

## Usage Notes

This occurrence report dataset includes both geographic and temporal information on the presence of *D. rotundus*. This information could be used to assess the distribution of the species retrospectively. Additionally, *D. rotundus* data could be coupled with environmental data to conduct ecological studies of the environmental tolerances of the species, landscape use, behavior, and prey availability. Future studies could also use this dataset to assess how *D. rotundus* distribution has changed over time and how distributional changes could be linked to climate and land cover change. Other potential applications of this dataset include the study of rabies reservoirs, which could aid in the understanding of rabies outbreaks. Epidemiological forecasting using *D. rotundus* data could serve to address gaps in current rabies prevention plans and could facilitate targeted social outreach and vaccination to vulnerable communities.

## Supplementary information

Supplementary Materials

## Data Availability

Data, metadata descriptions, and R code are available in the Figshare data repository for public access (accessible from: 10.6084/m9.figshare.15025296)^50^.

## Online-only Tables

**Online-only Table 1:** Data and metadata definitions.

Column Header	Definition
scientificName	Full scientific name and lowest level of taxonomic rank.
kingdom	The full scientific name of the kingdom in which the taxon is classified.
decimalLongitude	The geographic longitude (in decimal degrees, using the spatial reference system given in geodeticDatum) of the geographic center of Location. Positive values are east of the Greenwich Meridian, and negative values are west of it. Legal values lie between −180 and 180, inclusive.
decimalLatitude	The geographic latitude (in decimal degrees, using the spatial reference system given in geodeticDatum) of the geographic center of Location. Positive values are north of the Equator, and negative values are south of it.
geodeticDatum	The ellipsoid, geodetic datum, or spatial reference system (SRS) upon which the geographic coordinates are given in decimalLatitude and decimalLongitude as based.
countryCode	The standard code for the country in which the occurrence location occurs.
elevationInMeters	The elevation of the Location measured in meters (m).
individualCount	The number of individuals represented present at the time of the occurrence.
institutionID	An identifier for the institution having custody of the object(s) or information referred to in the record. The identifier used by the originating source of the occurrence.
family	The full scientific name of the family in which the taxon is classified.
taxonRank	The taxonomic rank of the most specific name in the scientificName.
coorditeUncertaintyInMeters	The horizontal distance (in meters) from the given decimalLatitude and decimalLongitude describing the smallest circle containing the whole of the Location.
stateProvince	The name of the next smaller administrative region than country (state, province, canton, department, region, etc.) in which the Location occurs.
locality	The specific description of the place the occurrence was reported. The lowest or most detailed level of geographic information.
year	The four-digit year in which the occurrence record occurred, according to the Common Era Calendar.
month	The integer month in which the occurrence record occurred.
day	The integer day of the month in which the occurrence record occurred.
basisOfRecord	The specific nature of the data record. The evidence on which the occurrence is based.
institutionCode	The name (or acronym) in use by the institution having custody of the object(s) or information referred to in the record.
datasetName	The name identifying the data set from which the record was derived.
Reference	Identifiers (publication, bibliographic reference, global unique identifier, URI) of literature associated with the occurrence.
sex	The sex of the biological individual(s) is represented in the occurrence. M = male, F = female.
lifeStage	The age class or life stage of the biological individual(s) at the time the occurrence was recorded.

The following table provides standardized descriptions of each occurrence metadata based on the Darwin Core archive format^51^. Each piece of metadata for each occurrence is organized and recorded under the listed column headers.

**Online-only Table 2:** Literary or published sources of occurrences.

Reference or Source	Number of Occurrence Reports
Abundes-Gallegos, J., Salas-Rojas, M., Galvez-Romero, G., Perea-Martinez, L., Obregon-Morales, C. Y., Morales-Malacara, J. B. & Nogueda-Torres, B. (2018). Detection of dengue virus in bat flies (Diptera: Streblidae) of common vampire bats, *Desmodus rotundus*, in Progreso, Hidalgo, Mexico. Vector-Borne and Zoonotic Diseases,18(1), 70-73.	1
Aguiar, L., Brito, D., & Machado, R. B. (2010). Do current vampire bat (*Desmodus rotundus*) population control practices pose a threat to Dekeyser’s nectar bat’s (*Lonchophylla dekeyseri*) long-term persistence in the Cerrado? Acta Chiropterologica, 12(2), 275–282.	1
Aguirre, L. F. (2002). Structure of a Neotropical savanna bat community. Journal of Mammalogy, 83(3), 775–784.	1
Aguirre, L. F., Mamani, C. J., Barbosa-Marquez, K., & Mantilla-Meluk, H. (2010). Lista actualizada de los murcielagos de Bolivia. Revista Boliviana de Ecologia y Conservacion Ambiental, 27(1), 1–7.	2
Almeida, E. O., Moreira, E. C., & Herrmann, G. P. (2002). Combat of *Desmodus rotundus rotundus* (E.E. Geoffroy, 1810) in the Cordisburgo and Curvelo carstic region, Minas Gerais, Brazil. Arquivo Brasileiro de Medicina Veterin ria e Zootecnia, 54(2), 117–126.	3
Alurralde, S. G., Barquez, R. M., & Diaz, M. M. (2017). New records of bats (Mammalia: Chiroptera) for a southern locality of the Argentine Yungas. Check List, 13(3), 2105.	3
Alviz, A. (2019). Fauna y Flora de Cinaruco - 2014–2016. v2.1. Parques Nacionales Naturales de Colombia. Dataset/Occurrence.	8
Andrade, F. A., Franta,. S., Souza, V. P., Barreto, M. S., & Fernandes, M. E. (2015). Spatial and temporal analysis of attacks by common vampire bats (*Desmodus rotundus*) on humans in the rural Brazilian Amazon basin. Acta Chiropterologica, 17(2), 393–400.	1
Anthony, S.J., Ojeda-Flores, R., Rico-Chávez, O., Navarrete-Macias, I., Zambrana-Torrelio, C.M., Rostal, M.K., Epstein, J.H., Tipps, T., Liang, E., Sanchez-Leon, M., Sotomayor-Bonilla, J., Aguirre, A.A., Ávila-Flores, R., Medellín, R.A., Goldstein, T., Suzán, G., Daszak, P., & Lipkin, W.I. (2013). Coronaviruses in bats from Mexico. The Journal of General Virology, 94(5), 1028.	1
Aragon, G., & Aguirre, M. (2014). Distribucion de murcielagos (Chiroptera) de la Region Tacna, Peru. Idesia, 32(1), 119–127.	1
Argibay, H.D., Orozco, M.M., Cardinal, M.V., Rinas, M.A., Arnaiz, M., Segura, C.M., Gurtler, R.E. (2016) First finding of Trypanosoma cruzi II in vampire bats from a district free of domestic vector-borne transmission in Northeastern Argentina. Parasitology. 143(11),1358–1368.	2
Asociación Becarios de Casanare - ABC (2009). Mamíferos laguna El Tinije, 58 records, provided by Ramírez, B. (Publisher, Resource Creator, Metadata Provider), Rodríguez, M. (Author), On line, http://ipt.sibcolombia.net/sib/resource.do?r = abc-2009-mamiferos, publicado el 25/10/2012.	1
Asprilla-Aguilar, A. A., Mantilla-Meluk, H., & Ortega, A. M. J. (2007). Analysis of the non-hematophagous bat species captured within the plan of eradication of *Desmodus rotundus* (E. Geoffroy, 1810) in the Colombian Biogeographic Choco. Revista Institucional Universidad Tecnologica del Choco Investigacion Biodiversidad y Desarrollo, 26(1), 42–48.	4
Avila-Gomez, E.S., Moreno, C.E., Garcia-Morales, R., Zuria, I., Sanchez-Rojas, G., Briones-Salas, M. (2015). Deforestation thresholds for phyllostomid bat populations in tropical landscapes in the Huasteca region, Mexico. Tropical Conservation Science. 8 (3), 646–661.	5
Bahlman, J. W., & Kelt, D. A. (2007). Use of olfaction during prey location by the common vampire bat (*Desmodus rotundus*) 1. Biotropica, 39(1), 147–149.	1
Bakker, K.M., Rocke, T.E., Osorio, J.E. *et al*. Fluorescent biomarkers demonstrate prospects for spreadable vaccines to control disease transmission in wild bats. Nature Ecology and Evolution, 3, 1697–1704 (2019).	1
Bakker, K.M., Rocke, T.E., Osorio, J.E., Abbott, R.C., Tello, C., Carrera, J.E., Valderrama, W., Shiva, C., Falcon, N., Streicker, D.G. (2019) Fluorescent biomarkers demonstrate prospects for spreadable vaccines to control disease transmission in wild bats. Nature Ecology and Evolution, 3(12), 1697–1704.	1777
Ballados-González, G. G., S. Sánchez-Montes, D. Romero-Salas, P. Colunga-Salas, R. Gutiérrez-Molina, L. León-Paniagua, I. Becker, M. L. Méndez-Ojeda, C. Barrientos-Salcedo, R. Serna-Lagunes, A. Cruz-Romero. 2018. Detection of pathogenic *Leptospira* species associated with phyllostomid bats (Mammalia: Chiroptera) from Veracruz, México. Transboundary and Emerging Diseases 65, 773–781.	2
Barrera Zambrano, V.A. (2017). Inventarios de flora y fauna en el piedemonte de los municipios Aguazul, Tauramena y Yopal del departamento de Casanare. v1.2. Asociación de Becarios del Casanare - ABC. Dataset/Occurrence.	9
Barrera Zambrano, V.A. (2018). Inventario y evaluación de fauna silvestre en el campo de exploración petrolera Niscota Sur. v2.1. Asociación de Becarios del Casanare - ABC. Dataset/Occurrence.	2
Barrera Zambrano, V.A. (2018). Línea base del medio biótico en los bosques dentro de la zona propuesta para Acuerdos de Conservación Voluntaria en la microcuenca de la Quebrada Aguazula - Rincón del Soldado Yopal. v2.0. Asociación de Becarios del Casanare - ABC. Dataset/Occurrence.	3
Barrera Zambrano, V.A. (2018): Línea base del medio biótico en los bosques para Acuerdos de Conservación Voluntaria en la microcuenca de la Quebrada Aguablanca El Morro Yopal. v2.2. Asociación de Becarios del Casanare - ABC. Dataset/Occurrence.	6
Barros, M. A. S., Morais Martins Gomes,C., Braga Figueiredo B. M., de Moura Júnior G. B., Fernandes dos Santos Ribeiro F., Marques Almeida Pessoa D., Ito F., & Bernard E.. (2017). Bats (Mammalia, Chiroptera) from the Nísia Floresta National Forest, with new records for the state of Rio Grande do Norte, northeastern Brazil. Biota Neotropica, 17(2), e20170351.	1
Becker, D.J., Bergner, L.M., Bentz A.B., Orton R.J., Altizer S., *et al*. (2018) Genetic diversity, infection prevalence, and possible transmission routes of *Bartonella* spp. in vampire bats. PLOS Neglected Tropical Diseases 12(9): e0006786.	9
Becker, D. J., Czirják, G. Á., Volokhov, D. V., Bentz, A. B., Carrera, J. E., Camus, M. S., Navara, K. J., Chizhikov, V. E., Fenton, M. B., Simmons, N. B., Recuenco, S. E., Gilbert, A. T., Altizer, S., & Streicker, D. G. (2018). Livestock abundance predicts vampire bat demography, immune profiles and bacterial infection risk. Philosophical transactions of the Royal Society of London. Series B, Biological sciences, 373(1745), 20170089.	10
Becker, D.J., Nachtmann, C., Argibay, H.D., Botto, G., Escalera-Zamudio, M., Carrera, J.E., Tello, C., Winiarski, E., Greenwood, A.D., Mendez-Ojeda, M.L., Loza-Rubio, E., Lavergne, A., de Thoisy, B., Czirjak, G.A., Plowright, R.K., Altizer, S., Streicker, D.G. (2019). Leukocyte profiles reflect geographic range limits in a widespread neotropical bat. Integrative and Comparative Biology, 59(5),1176–1189.	33
Bejarano-Bonilla, D. A., Yate-Rivas, Alexander & Bernal-Bautista, M. H. (2007). Bat diversity and distribution along an altitudinal transect in the Tolima region of Colombia. Caldasia, 29(2), 297–308.	1
Bergner, L.M., Orton, R.J., Benavides, J.A., Becker, D.J., Tello, C., Biek, R., & Streicker, D.G. (2020). Demographic and environmental drivers of metagenomic viral diversity in vampire bats. Molecular Ecology, 29, 6–39.	8
Bernard, E. (2001). Species list of bats (Mammalia, Chiroptera) of Santarem area, Para state, Brazil. Revista Brasileira de Zoologia, 18(2), 455–463.	1
Bernard, E. &Fenton, B. (2007). Bats in a fragmented landscape: Species composition, diversity and habitat interactions in savannas of Santarem, Central Amazonia, Brazil. Biological Conservation, 134(3),332–343.	1
Bichuette, ME; Gimenez, ED; Arnone, IS; & Trajano, E (2018) An important site for conservation of bats in Brazil: Passa Tres cave, Sao Domingos karst area, with an updated checklist for Distrito Federal (DF) and Goias state. Subterranean Biology, 28, 39–51.	1
Bobrowiec, P. E. D., Lemes, M. R., & Gribel, R. (2015). Prey preference of the common vampire bat (*Desmodus rotundus*, Chiroptera) using molecular analysis. Journal of Mammalogy, 96(1), 54–63.	3
Bolívar‐Cimé, B., Cuxim‐Koyoc, A., Reyes‐Novelo, E., Morales‐Malacara, J.B., Laborde, J. and Flores‐Peredo, R. (2018), Habitat fragmentation and the prevalence of parasites (Diptera, Streblidae) on three Phyllostomid bat species. Biotropica, 50, 90–97.	2
Bruna da Silva, X., Carvalho, W.D., Dias, D., Tabosa, L.O., Santos, C., & Esbérard, C.E.L. (2018). Bat richness (Mammalia: Chiroptera) in an area of montane Atlantic Forest in the Serra da Mantiqueira, state of Minas Gerais, southeast Brazil. Biota Neotropica, 18(2), e20170496.	1
Camargo, A., Olivera, R., Santiago, M., &González, J. (2018). Genetic relatedness of Desmodus rotundus from northern Uruguay with populations from the remainder of its distribution range. *Boletín de la Sociedad Zoológica del Uruguay, 27*(1), 14–18.	1
Carrillo-Araujo, M., Tas, N., Alcantara-Hernandez, R.J., Gaona, O., Schondube, J.E., Medellin, R.A., Jansson, J.K., &Falcon, L.I. (2015) Phyllostomid bat microbiome composition is associated to host phylogeny and feeding strategies. Frontiers in Microbiology, 6, 447.	1
Carter, G.G., Farine, D.R., Crisp, R.J., Vrtilek, J.K., Ripperger, S.P., &Page, R.A., Development of new food-sharing relationships in vampire bats. Current Biology, 30(7), 307–309.	2
Castilla, C. M., Torres, R., and Díaz, M. M. (2013). Murciélagos de la provincia de Córdoba, Argentina: riqueza y distribución. Mastozoología Neotropical, 20(2), 243–254.	12
Castilla, M. C. (2018). Diagnóstico etno-zoológico y biogeográfico del ensamble de murciélagos del dique de escaba: Implicancias para su conservación. Ph.D. Thesis. Universidad Nacional de Córdoba.	14
	11
Castillo, L.S., Cardona, D. (2018). Registro de murciélagos del Cañón del Chicamocha, Santander (2013–2014). v1.0. Fundación Chimbilako. Dataset/Occurrence.	3
Castro M.M., Kim B., Hill E., Fialho M.C., Puga L.C., Freitas M.B., Breton S., Machado-Neves M. (2017). The expression patterns of aquaporin 9, vacuolar H^+^-ATPase, and cytokeratin 5 in the epididymis of the common vampire bat. Histochemistry Cell Biology. 147(1), 39–48.	1
Cavelier Franco, I. (2020). Proyecto COL88611 para la conservación y uso sostenible de ecosistemas secos. v2.1. Patrimonio Natural Fondo para la Biodiversidad y Áreas Protegidas Patrimonio Natural. Dataset/Occurrence.	48
Cimé, B., R. Flores-Peredo, S. A. García-Ortíz, R. Murrieta-Galindo, J. Laborde. (2019). Influence of landscape structure on the abundance of Desmodus rotundus (Geoffroy 1810) in northeastern Yucatan, Mexico. Ecosistemas y Recursos Agropecuarios, 6(17), 261–271.	2
Corporación Autónoma Regional del Valle del Cauca - CVC y Universidad del Valle (2018). Análisis de Integridad Biológica en el Parque Natural Regional Páramo del Duende. Convenio 108 de 2017. Universidad del Valle. Disponible en:	1
Corporación Autónoma Regional del Valle del Cauca - CVC y Universidad del Valle (2018). Análisis de Integridad Biológica en el Distrito Regional de Manejo Integrado RUT Nativos. Convenio 108 de 2017. Universidad del Valle.	2
Correia Dos Santos L., Vidotto O., Dos Santos N.J.R., Ribeiro J., Pellizzaro M., Dos Santos A.P., Haisi A., Wischral Jayme Vieira T.S., de Barros Filho I.R., Cubilla M.P., Araujo J.P. Junior, da Costa Vieira R.F., Ullmann L.S., & Biondo A.W. (2020). Hemotropic mycoplasmas (hemoplasmas) in free-ranging bats from Southern Brazil. Comparative Immunology, Microbiology, and Infectious Diseases, 69, e101416.	1
Costa, L. M., & Esbérard, C. E. (2011). *Desmodus rotundus* (Mammalia: Chiroptera) on the southern coast of Rio de Janeiro state, Brazil. *Brazilian Journal of Biology, Revista Brasileira de Biologia*, 71(3), 739–746.	21
da Rocha, P.A., Ruiz-Esparza, J., Ribeiro, Adauto de Souza, Ferrari, S.F. (2015). Species diversity and seasonal variation in the composition of a bat community in the semi-arid Brazilian caatinga. Acta Scientiarum Biological Sciences, 37(2), 197–203.	8
Dantas Torres F., Valença C., Andrade Filho G.V. (2005). First record of *Desmodus rotundus* in urban area from the city of Olinda, Pernambuco, Northeastern Brazil: a case report. Rev Inst Med Trop Sao Paulo, 47(2),107–8.	1
de Castro Ferreira E., Pereira A.A.S., Silveira M., Margonari C., Marcon G.E.B., de Oliveira França A., Castro L.S., Bordignon M.O., Fischer E., Tomas W.M., Dorval M.E.C., Gontijo C.M.F. (2017). *Leishmania* (V.) *braziliensis* infecting bats from Pantanal wetland, Brazil: First records for *Platyrrhinus lineatus* and *Artibeus planirostris*. Acta Tropica. 172, 217–222.	1
• de Castro, Mariana Moraes, Wagner Gonzaga Gontalves, Stephanie Assef Millen Valente Teixeira, Maria do Carmo Queiroz Fialho, Felipe Couto Santos, Jerusa Maria Oliveira, Jose Eduardo Serrio, and Mariana Machado-Neves. (2017). Ultrastructure and morphometric features of epididymal epithelium in *Desmodus rotundus*. Micron 102, 35–43.	1
Delpietro, H.A., Russo, R.G., Carter, G.G., Lord, R.D., & Delpietro, G.L. (2017). Reproductive seasonality, sex ratio and philopatry in Argentina’s common vampire bats. Royal Society Open Science, 4, e160959	1617
Dornelles, Guilherme D. P., Graciolli, Gustavo, Odon, Anderson, and Bordignon, Marcelo O. (2017). Infracommunities of Streblidae and Nycteribiidae (Diptera) on bats in an ecotone area between Cerrado and Atlantic Forest in the state of Mato Grosso do Sul. Iheringia. Série Zoologia, 107, e2017044	1
De Rodrigues Coelho E., A. Pereira Paglia, A. Barbosa Viana-Junior, L. A. Dolabela Falcão, & G. B. Ferreira. (2018). Species richness, abundance and functional diversity of a bat community along an elevational gradient in the Espinhaço mountain range, southeastern Brazil. Acta Chiropterologica, 20(1), 129–138.	7
Escuela de Biología, Universidad Industrial de Santander, (2014). Colección Mastozoológica de la Universidad Industrial de Santander. 732 registros, aportados por Gregorio Moreno (proveedor de contenidos) y Mauricio Torres (procesador, custodio, proveedor de metadatos, publicador, editor), En línea, publicado el 11/09/2014 (actualizado el 03/03/2015).	6
Falcão L.A.D, Espírito-Santo M.M., Fernandes G.W., Paglia A.P. (2018). Effects of habitat structure, plant cover, and successional stage on the bat assemblage of a tropical dry forest at different spatial scales. *Diversity*. 10(2), 41–43.	5
Farfan Espinosa E.F., Valencia Mazo J.D., Moreno Oliveros A., Velásquez Valencia, A. (2021). Colección de mamíferos del Museo de Historia Natural de la Universidad de la Amazonia (UAM-M). v2.2. Universidad de la Amazonia. Dataset/Occurrence.	2
Ferraz, C., Achkar, S. M., and Kotait, I. (2007). First report of rabies in vampire bats (*Desmodus rotundus*) in an urban area, Ubatuba, Sao Paulo State, Brazil. Revista do Instituto de Medicina Tropical de Sao Paulo, 49(6), 389–390.	1
Fierro Calderon, K. (2018). Caracterización de Flora y Fauna en la cuenca del río Calamar, municipios de Bolívar y Trujillo, Valle del Cauca. v1.0. Asociación para el estudio y conservación de las aves acuáticas en Colombia. Dataset/Occurrence.	2
Fischer, E., Ferreira Santos, C., Alves da Cunha Carvalho, L.F., Camargo, G., Leme da Cunha, N., Silveira, M., Bordignon, M.O., &de Lima Silva, C. (2015). Bat fauna of Mato Grosso do Sul, southwestern Brazil. Biota Neotropica, 15(2), e20140066.	3
Franco Filho L.C., Barata R.R., Cardoso J.F., Massafra J.M.V, Lemos P.D.S., Casseb L.M.N., Cruz A.C.R., & Nunes M.R.T. (2019). Metagenomic analysis of samples from three bat species collected in the Amazon rain forest. Microbiology Resource Announcment, 8(2), e01422–18.	1
Fundación Omacha, Instituto de Investigación de Recursos Biológicos Alexander von Humboldt (2015). Caracterización de fauna y flora para el establecimiento de límites funcionales de humedales en tres ventanas piloto: Ciénaga de la Virgen, Ciénaga Zapatosa y Paz de Ariporo - Hato Corozal. 3027 registros, aportados por: Lasso, C. (Contacto del recurso), Trujillo, F. (Creador del recurso), Velásquez, J. (Proveedor de metadatos). Versión 5.1.	1
Fundación Reserva Natural La Palmita Centro de Investigación, Instituto de Investigación de Recursos Biológicos Alexander von Humboldt (2015). Caracterización biotica para la conservación de especies amenazadas en el área de influencia del Oleoducto Bicentenario, departamentos de Arauca y Casanare, Colombia. 3034 registros, aportados por: Vieira, M-I. (Contacto del recurso), Mora-Fernández, C. (Creador del recurso, Autor), Rodríguez-Posada, M. E. (Autor), Trujillo, W. (Autor), Henao, M. (Autor), Zamudio, J. (Autor), Preciado, V. (Autor), Blanco, A. (Autor), Romero, H. (Autor), Carantón, D. (Autor), Restrepo, M. (Autor), Fernández, C. (Autor), Gutierrez, D. (Autor). Versión 7.0.	2
Garcia, F.J., Araujo-Reyes, D., Vasquez-Parra, O., Brito, H., & Machado, M. (2015). Murciélagos (Mammalia: Chiroptera) asociados con una cueva en el parque nacional yurubí, sierraParque Nacional Yurubí, Sierra de aroaAroa, estado yaracuy, Yaracuy, Venezuela. Caldasia, 37(2), 381–391.	1
Gardner, A. L. (2008). Mammals of South America, Volume 1: Marsupials, Xenarthrans, Shrews and Bats.The University of Chicago Press, Chicago. 690pp.	2
Pignaton Gnocchi, A., &Srbek-Araujo, A. C.. (2017). Common Vampire Bat (*Desmodus rotundus*) feeding on Lowland Tapir (*Tapirus terrestris*) in an Atlantic Forest remnant in southeastern Brazil. Biota Neotropica, 17(3), e20170326.	2
Gonzalez, C. (2019). Murcielagos de Paraguay, Biosfera, Publicaciones Del Comite Espanol del Programa Hombre y Biosfera Red Iberomab Unesco. 302pp	19
Gorresen, P. M., &Willig, M. R. (2004). Landscape responses of bats to habitat fragmentation in Atlantic forest of Paraguay. Journal of Mammalogy, 85(4), 688–697.	1
Grattarola F., Botto G., da Rosa I., Gobel N., González E., González J., Hern ndez D., Laufer G., Maneyro R., Martinez-Lanfranco J., Naya D., Rodales A., Ziegler L., & Pincheira-Donoso D. (2019) Biodiversidata: An open-access biodiversity database for Uruguay. Biodiversity Data Journal 7: e36226.	67
Gregorin, R., Bernard, E., Lobao, K.W., Oliveira, L.F., Machado, F.S., Gil, B.B., & Tavares, V.D. (2017). Vertical stratification in bat assemblages of the Atlantic Forest of south-eastern Brazil. Journal of Tropical Ecology, 33(5), 299–308.	2
Hayes M.A., &Piaggio A.J. (2018). Assessing the potential impacts of a changing climate on the distribution of a rabies virus vector. PLoS ONE 13, e0192887.	1145
Hernández-Pérez, E.L., Castillo-Vela, G., García-Marmolejo, G., Sanvicente López, M., &Reyna-Hurtado, R. (2019). Wild pig (*Sus scrofa*) as prey of the Common Vampire Bat (*Desmodus rotundus*). Therya, 10 (2), 195–199.	1
Huguin, M., Arechiga-Ceballos, N., Delaval, M., Guidez, A., de Castro, I.J., Lacoste, V., Salmier, A., Setién, A.A., Silva, C.R., Lavergne, A., & de Thoisy, B. (2018). How social structure drives the population dynamics of the common vampire bat (*Desmodus rotundus*, Phyllostomidae). Journal of Heredity, 109(4), 393–404.	5
Ingala, M.R., Becker, D.J., Bak Holm, J., Kristiansen, K., & Simmons, N.B. (2019). Habitat fragmentation is associated with dietary shifts and microbiota variability in common vampire bats. Ecology and Evolution, 9, 6508–6523.	2
Instituto de Investigación de Recursos Biológicos Alexander von Humboldt (2016). Tejidos de quirópteros de tres localidades del Caribe Colombiano. 240 tejidos, aportados por: Mantilla-Meluk, H. (Creador del recurso). Versión 1.1.	24
Instituto de Investigación de Recursos Biológicos Alexander von Humboldt (2015). Caracterización biológica de la ventana de biodiversidad Municipio de Filandia, Quindío, 864 registros, aportados por: Palacio, R.D. D., Meluk, H., Vergara, M., Ortiz-Movliav, C., Echeverri, C., Lizarazo, J., Moreno, J.S., Vergara, L.F., Hiniestroza, T., Ríos, J.A., Cataño, A., Guevara-Molina, C., Versión 1.1.	1
Instituto de Investigación de Recursos Biológicos Alexander von Humboldt (2014). Mamíferos pequeños en el municipio de Medina, Cundinamarca - Proyecto Colombia Bio. 44 registros, aportados por: González, M. (Contacto del recurso), Cruz Moncada, M. (Creador del recurso y Proveedor de metadatos).	10
Instituto de Investigación de Recursos Biológicos Alexander von Humboldt (2018). Tejidos de ejemplares colectados en el Páramo de Chingaza-Proyecto Colombia Bio. 1215 registros, aportados por: González, M. (Creador del recurso, Contacto del recurso), Infrastructura Institucional de datos. (Proveedor de metadatos).	10
Instituto de Investigación de Recursos Biológicos Alexander von Humboldt (2016). Registros de quirópteros de tres localidades del caribe colombiano. 168 registros, aportados por: Mantilla-Meluka, H. (Creador del recurso). Versión 1.1.	25
Instituto de Investigación de Recursos Biológicos Alexander von Humboldt, Biotica Consultores Ltda. (2014). Fauna y flora de la cuenca media del río Lebrija en Rionegro, Santander. 761 registros, aportados por: Barriga, J. (Contacto del recurso), Dueñas, A. (Creador del recurso, Proveedor de metadatos). Versión 1.1.	1
Instituto de Investigación de Recursos Biológicos Alexander von Humboldt (2015). Contexto Biótico y Socio-económico de la parte norte de la Ciénaga de Zapatosa. 1098 registros, aportados por: Galvis, G. (Autor, Contacto del recurso, Creador del recurso y Proveedor de metadatos), Morelo, L. A. (Autor), Carvajal, E. (Autor), Ardila, M. (Autor), Mantilla, H (Autor). Versión 4.0.	1
Isagen, S.A. (2009). Registros biológicos de especies de fauna vertebrada terrestre en las centrales de San Carlos y Jaguas - 2009, 4290 registros, aportados por Pérez, C. (Publicador, Creador del Recurso, Proveedor de los Metadatos), Muñoz-Escobar, E. (Proveedor de Contenido), Gallo, S.M. (Proveedor de Contenido), Peña, A.F. (Proveedor de Contenido), Palacio-V, J.A. (Proveedor de Contenido), Duque, V.M. (Editor), En linea, http://ipt.sibcolombia.net/sib/resource.do?r = isagen-2008-46-2978, publicado el 07/12/2012.	2
Jayat, J. P., and Ortiz, P. E. (2010). Mamiferos del pedemonte de Yungas de la Alta Cuenca del Rio Bermejo en Argentina: Una linea de base de diversidad. Mastozoolog ia Neotropical, 17(1), 69–86.	8
Jimenez Ortega, A.M. (2014). Colección teriológica de la Universidad Tecnológica del Chocó. v5.3. Universidad Tecnológica del Chocó. Dataset/Occurrence.	10
Jones, M.F., and Hasiotis, S.T. (2018). Terrestrial behavior and trackway morphology of neotropical bats. Acta Chiropterologica, 20(1), 229–250.	1
Kraker-Castañeda, C., Echeverría-Tello, J. (2012). Riqueza de Especies y Variabilidad Trófica de Murciélagos en Zonas de Riesgo de Rabia de Origen Silvestre en Izabal, Guatemala. *Therya*, 3(1), 87–99.	4
Lee, D.N., Pape, M., Van Den Bussche, R.A. (2012). Present and potential future distribution of common vampire bats in the Americas and the associated risk to cattle. PLoS ONE, 7, e42466.	12224
Leigue Dos Santos, L., Montiani-Ferreira, F., Lima, L., Lange, R., de Barros Filho, I.R. (2014). Bacterial microbiota of the ocular surface of captive and free-ranging microbats: *Desmodus rotundus*, *Diameus youngi* and *Artibeus lituratus*. *Veterinary Ophthalmology*, 17(3), 157–61.	1
Lima, C.S., Varzinczak, L.H. and Passos, F.C. (2017). Richness, diversity and abundance of bats from a savanna landscape in central Brazil. *Mammalia*, 81(1), 33–40.	2
López, H., Raz, L., Agudelo, H. (2020). Colección de Mamíferos del Instituto de Ciencias Naturales (ICN-MHN-Ma). v2.9. Universidad Nacional de Colombia. Dataset/Occurrence.	65
Lourenço, E. C., Costa, L. M., Silva, R. M., & Esbérard, C. E. (2010). Bat diversity of Ilha da Marambaia, Southern Rio de Janeiro State, Brazil (Chiroptera, Mammalia). *Brazilian journal of biology, Revista brasleira de biologia*, 70(3), 511–519.	5
Mantilla Meluk, H., Herrera Collazos, E. (2019). Murciélagos de las sabanas inundables de las cuencas de los ríos Bita, Manacacías y Cravo Sur - SULU II. v1.1. Fundación Omacha & World Wildlife Fund Colombia (WWF). Dataset/Occurrence.	2
Martínez‐Fonseca, J.G., Chávez‐Velásquez, M., Williams‐Guillen, K., & Chambers, C.L. (2020). Bats use live fences to move between tropical dry forest remnants. *Biotropica*, 52, 5–10.	1
Martins F.F., Puga C.C., Beguelini M.R., Morielle-Versute E., Vilamaior P.S., & Taboga S.R. (2015). Comparative analysis of the male reproductive accessory glands of bat species from the five Brazilian subfamilies of the family Phyllostomidae (Chiroptera). *Journal of Morphology*. 276(4), 470–80.	1
Martins, M. A., De Carvalho, W. D., Dias, D., Franta, D. D. S., De Oliveira, M. B., & Peracchi, A. L. (2015). Bat species richness (Mammalia, Chiroptera) along an elevational gradient in the Atlantic Forest of Southeastern Brazil. Acta Chiropterologica, 17(2), 401–409.	1
Mcdonough, M.M., Ferguson, A.W., Ammerman, L.K., Granja-Vizcaino, C., Burneo, S.F., and Baker, R.J. (2011). Molecular verification of bat species collected in ecuadorEcuador: Results of a country-wide survey. Papers Of The Museum Of Texas Tech University 301,1–28.	4
Mendes, P. Consequências da perda e fragmentação de habitat em morcegos. (2015). Ph.D. Thesis. Universidade Federal de Goiás, Goiânia, 2015.	15
Mendes, P., With, K.A., Signorelli, L., and De Marco Jr, P. (2017). The relative importance of local versus landscape variables on site occupancy in bats of the Brazilian Cerrado. Landscape Ecology, 32, 745–762.	67
Menezes, L.F., Duarte, A.C., Contildes, M.D., Peracchi, A.L. (2015). Comparison of chiropterofauna at forested and open areas of “RPPN FazendaBom Retiro”, Rio de Janeiro, Brazil. Iheringia, Série Zoologia, Porto Alegre, 105(3):271–275.	1
Mialhe, P. J. (2013). Characterization of *Desmodus rotundus* (E. Geoffroy, 1810) (Chiroptera, Phyllostomidae) shelters in the Municipality of Sao Pedro-SP. Brazilian Journal of Biology, 73(3), 521–526.	1
Mialhe, P. J. (2014). Preferential prey selection by *Desmodus rotundus* (E. Geoffroy, 1810, Chiroptera, Phyllostomidae) feeding on domestic herbivores in the municipality of Sao Pedro-SP. Brazilian Journal of Biology, 74(3), 579–584.	1
Morais, D. B., Puga, L. C. H. P., de Paula, T. A. R., Freitas, M. B. D., & da Matta, S. L. P. (2017). The spermatogenic process of the common vampire bat *Desmodus rotundus* under a histomorphometric view. PLoS ONE, 12(3), e0173856.	1
Morales Martínez, D., Herrera-Collazos, E.E. (2017). Murciélagos observados en la transición Andino Amazónica del departamento del Caquetá - Proyecto Colombia Bio. v1.3. Instituto Amazónico de Investigaciones Científicas Sinchi. Dataset/Occurrence.	17
Morales, D., Herrera-Collazos, E.E. (2017). Mamíferos colectados en la transición Andino-Amazónica del departamento del Caquetá - Proyecto Colombia Bio. v2.5. Instituto Amazónico de Investigaciones Científicas Sinchi. Dataset/Occurrence.	1
Muylaert, R. D. L., Stevens, R. D., Esb‚rard, C. E., Mello, M. A., Garbino, G. S., Varzinczak, L. H., Faria, D., Weber, M.d.M., Kerches Rogeri, P., Regolin, A.L., Oliveira, H.F.M.d., Costa, L.d.M., Barros, M.A.S., Sabino‐Santos, G., Jr, Crepaldi de Morais, M.A., Kavagutti, V.S., Passos, F.C., Marjakangas, E.‐L., Maia, F.G.M., Ribeiro, M.C. and Galetti, M. (2017). Atlantic bats: A data set of bat communities from the Atlantic forests of South America. Ecology, 98(12), 3227–3227.	92
Muylaert, R.L., Stevens, R.D. and Ribeiro, M.C. (2016), Threshold effect of habitat loss on bat richness in cerrado‐forest landscapes. Ecological Applications, 26, 1854–1867.	1
Nunez, H. A., & de Viana, M. L. (1997). Estacionalidad reproductiva en el vampiro comon *Desmodus rotundus* (Chiroptera, Phyllostomidae) en el Valle de Lerma (Salta, Argentina). Revista de Biologia Tropical, 1231–1235.	1
Orozco, M.M., Enriquez, G.F., Cardinal, M.V., Piccinali, R.V., Gürtler, R.E. (2016). A comparative study of *Trypanosoma cruzi* infection in sylvatic mammals from a protected and a disturbed area in the Argentine Chaco. Acta Tropica. 155, 34–42.	1
Ossa, G., Forero, L., Novoa, F., and Bonacic, C. (2015). Caracterizacion morfologica y bioacustica de los murcielagos (Chiroptera) de la Reserva Nacional Pampa de Tamarugal. Biodiversidata, 3, 21–29.	1
Owen, J.G. &Giron, L. (2012). Revised checklist and distributions of land mammals of El Salvador. Museum of Texas Tech University, 310, 8.	4
Parez-Rivero, J. J., Parez-Martinez, M., &Aguilar-Setin, A. (2014). Histometric analysis of vampire bat (*Desmodus rotundus*) testicles treated with coumestrol by oral route. Journal of Applied Animal Research, 42(2), 208–212.	1
Pinto, C. M. (2009). Genetic diversity of the common vampire bat Desmodus rotundus in Ecuador: testing cross-Andean gene flow. Ph.D. Thesis.	18
Portfors, C.V., Fenton, M.B., de S. Aguiar, L.M., Baumgarten, J.E., Vonhof, M.J., Bouchard, S., de Faria, D.M., Pedro, W.A., Rauntenbach, N.I.L, & Zortea, M. (2000). Bats from Fazenda Intervales, Southeastern Brazil: Species account and comparison between different sampling methods. Revista brasileiraBrasileira de Zoologia 17(2), 533–538.	1
Ramírez-Chaves, H.E., Mejía Fontecha, I.Y., Velasquez, D., Castaño, D., Ocampo, D. (2020). Colección de Mamíferos (Mammalia) del Museo de Historia Natural de la Universidad de Caldas, Colombia. v2.4. Universidad de Caldas. Dataset/Occurrence.	23
Renata M. Pereira Freitas, Jerusa M. Oliveira, David L. Justinico Castro, Mariaurea Matias Sarandy, Reggiani Vilela Gonçalves, Mariella Bontempo Freitas (2020). The antioxidant status of three neotropical bat species with different feeding habits. Acta Chiropterologica, 21(2), 395–402.	1
Reyes-Amaya, N., &Jerez, A. (2013). Postnatal cranial ontogeny of the common vampire bat *Desmodus rotundus* (Chiroptera: Phyllostomidae). Chiroptera Neotropical, 19(2), 1198–1211.	1
Rick, A. M. (1968). Notes on bats from Tikal, Guatemala. Journal of Mammalogy, 49(3), 516–520.	1
Ripperger, S., Carter, G., Duba, N., Koelpin, A., Cassens, B., Kapitza, R., Josic, D., Berrio-Martinez, J., Page, R.A., Mayer, F. (2019). Vampire Bats that cooperate in the lab maintain their social networks in the wild. Current Biology, 29(23), 4139–4144.	2
Rocha, A.D., &Bichuette, M.E. (2016). Influence of abiotic variables on the bat fauna of a granitic cave and its surroundings in the state of São Paulo, Brazil. Biota Neotropica, 16(3), e20150032.	1
Rocha, F., Ulloa-Stanojlovic, F.M., Rabaquim, V.C.V., Fadil, P., Pompei, J.C., Brandao, P.E., & Dias, R.A. (2020). Relations between topography, feeding sites, and foraging behavior of the vampire bat, *Desmodus rotundus. Journal of Mammalogy*, 1(1),164–171.	9
Rojas, A., Jimanez, A., Vargas, M., Zumbado, M., & Herrero, M. V. (2008). Ectoparasites of the common vampire bat (*Desmodus rotundus*) in Costa Rica: parasitism rates and biogeographic trends. Mastozoologia Neotropical, 15(2), 181–187.	12
Romero-Barrera, C., Osorio-Rodriguez, A., & Juárez-Agis, A. (2021). Distribución, abundancia, control y registros de casos de murciélagos vampiro, *Desmodus rotundus* (E. Geoffroy), infectados de rabia en ambientes pecuarios de Guerrero, México. Acta Agrícola y Pecuaria, 7, e0071005.	178
Romero-Nava, C., Leon-Paniagua, L., and Ortega, J. (2014). Microsatellites loci reveal heterozygosis and population structure in vampire bats (*Desmodus rotundus*) (Chiroptera: Phyllostomidae) of Mexico. Revista de biologia tropicalBiologia Tropical, 62(2), 659–669.	14
Salmier A., de Thoisy B., Crouau-Roy B., Lacoste V., Lavergne A. (2016). Spatial pattern of genetic diversity and selection in the MHC class II DRB of three Neotropical bat species. BMC Evolutionary Biology, 16(1), 229.	36
Salmier A., Tirera S., de Thoisy B., Franc A., Darcissac E., Donato D., Bouchier, C., Lacoste, V., and Lavergne, A. (2017) Virome analysis of two sympatric bat species (*Desmodus rotundus* and *Molossus molossus*) in French Guiana. PLoS ONE 12(11): e0186943.	2
Salmón-Mulanovich, G., Vásquez, A., Albújar, C., Guevara, C., Laguna-Torres, A., Salazar, M. and Montgomery, J. M. (2009). Human rabies and rabies in vampire and nonvampire bat species, southeastern Peru, 2007. Emerging Infectious Diseases, 15(8), 1308–1311.	2
Sanchez, F., J. Alvarez, Clara Ariza, and A. Cadena. (2007). Bat assemblage structure in two dry forests of Colombia: Composition, species richness, and relative abundance. Mammalian Biology 72(2) 82–92.	1
Sanchez-Cordero, V., Botello, F., Magana-Cota, G., & Iglesias, J. (2011). Vampire bats, *Desmodus rotundus*, feeding on white-tailed deer, *Odocoileus virginianus*. Mammalia, 75(1), 91–92.	2
Sánchez-Hernández, C., Zalapa, S.S., Guerrero, S., Romero-Almaraz, M., Sil-Berra, L.M., and Schnell, G.D. (2018). Ocular lesions and diseases in bats from Jalisco and Oaxaca, Mexico. Acta Chiropterologica, 20(2), 485–492.	5
Sandoval, M., Sánchez, M., & Barquez, R.M. (2010). Mammalia, Chiroptera Blumenbach, 1779: New locality records, filling gaps, and geographic distribution maps from northern Argentina. Check List, 6, 64–67.	8
Santana, A., Carvalho, C., Alves, A.J, Pedro, W., and Queiroz, L. (2018). Population of hematophagous bats in the Andradina region, São Paulo: Roost and control characterization. Acta Veterinaria Brasilica. 12. 62–70.	9
Santos, N., Fagundes, V., Yonenaga Yassuda, Y., & De Souza Jose, M. (2001). Comparative karyology of Brazilian vampire bats *Desmodus rotundus* and *Diphylla ecaudata* (Phyllostomidae, Chiroptera): Banding patterns, base specific fluorochromes and fish of ribosomal genes. Hereditas, 134(3), 189–194.	3
Santos-Moreno, A., Aldape-López, C.T., Benítez-Díaz, C., and Martínez-Coronel, M. (2016). Ampliación del límite superior de distribución altitudinal de tres especies de mamíferos en Oaxaca, México. Revista Mexicana de Biodiversidad, 87(1), 267–269.	1
Shapiro, J. T., and Bordignon, M. O. (2014). Bat (Chiroptera) assemblages in three Cerrado fragments of Mato Grosso do Sul, southwestern Brazil. Check List, 10(6), 1380–1386.	4
Sierra Leal, J.A., Sarmiento, J., Carrero, D., Acevedo, A., Rodríguez, P. (2020). Colección de Zoología General de la Universidad de Pamplona. v2.4. Universidad de Pamplona. Dataset/Occurrence.	1
Siles, L., Penaranda, D., Perez-Zubieta, J. C., and Barboza, K. (2005). Los murci‚lagos de la ciudad de Cochabamba. Revista Boliviana de Ecologia y Conservacion Ambiental, 18, 51–64.	1
Solari, S. (2017). Colección Teriológica Universidad de Antioquia. v1.1. Universidad de Antioquia. Dataset/Occurrence.	1
Souza A.C.F., Santos F.C., Bastos D.S.S., Sertorio M.N., Teixeira J.P.G., Fernandes K.M., and Machado-Neves, M. (2018) Reproductive functions in *Desmodus rotundus*: A comparison between seasons in a morphological context. PLoS ONE 13(10): e0205023.	1
Streicker, D.G., Winternitz, J.C., Satterfield, D.A., Condori-Condori, R.E., Broos, A., Tello, C., Recuenco, S., Velasco-Villa, A., Altizer, S., Valderrama, W. (2016). Host-pathogen evolutionary signatures reveal dynamics and future invasions of vampire bat rabies. Proceedings of the National Academy of Sciences, 113, 10926–10931.	10
Streicker, D.G., Recuenco, S., Valderrama, W., Benavides, J.G., Vargas, I., Pacheco, V., Condori, R.E.C., Montgomery, J., Rupprecht, C.E., Rohani, P. and Altizer, S. (2012). Ecological and anthropogenic drivers of rabies exposure in vampire bats: Implications for transmission and control. Proceedings of the Royal Society B: Biological Sciences, 279, 3384–3392.	19
Tirira, D. G. and S. F. Burneo (eds.). (2012). Investigacion y conservacion sobre murcielagos en el Ecuador Pontificia Universidad Catolica del Ecuador, Fundacion Mamiferos y Conservacion y Asociacion Ecuatoriana de Ecuador. Quito Mastozoologia. Publicación especial sobre los mamíferos del Ecuador 9, 179–182.	3
Tlapaya-Romero, L., Ibanez-Bernal, S., Santos-Moreno, A. (2019) New records of bat flies (Diptera: Streblidae) in Oaxaca, Mexico. Inicio 90, e2894.	1
Torquetti C.G., Silva M.X., Talamoni S.A. (2017) Differences between caves with and without bats in a Brazilian karst habitat. Zoologia, 34, 1-7.	1
Trujillo-Pahua, L., and Ibáñez-Bernal, S. (2019). New geographical records of bat flies (Diptera: Streblidae) associated with phyllostomid bats (Chiroptera: Phyllostomidae) in the West Highlands of Mexico. Journal of Medical Entomology. 56(1), 18–28.	3
Turcios-Casco, M.A., Mazier, D.I.O., Orellana, J.A.S., Ávila-Palma, H.D., Trejo, E.J.O. (2019). Two caves in western Honduras are important for bat conservation: first checklist of bats in Santa Bárbara. Subterranean Biology, 30, 41–55.	3
Ueti, A., Pompeu, P.S., Ferreira, R.L. (2015) Asymmetry compensation in a small vampire bat population in a cave: A case study in Brazil. Subterranean Biology, 15, 57–67.	1
Uieda, W. (2001). Behavior of an albino vampire bat, *Desmodus rotundus* (E. Geoffroy) (Chiroptera, Phyllostomidae), in captivity. Revista Brasileira de Zoologia, 18(2), 641–644.	1
Universidad de Córdoba, Instituto de Investigaciones Biológicas Alexander von Humboldt (2018). Vertebrados de los humedales de La Mojana, Colombia. 2838 registros. Aportados por: Jaramillo, U. (Contacto del recurso), Linares-Arias, J.C. (Creador del recurso, Investigador Principal), Carrillo-fajardo, M.Y. (Proveedor del recurso, Editor), Ortega-León, A.M. (Editor), Ballesteros-Correa, J. (Editor), González-Charrasquiel, C.M. (Editor), Vergara-Doria, L.E. (Proveedor de contenido), Prioló-Espitia, M.C. (Proveedor de contenido), Martínez, J. (Proveedor de contenido), Berrocal, M.E. (Proveedor de contenido), Pérez, G.P (Proveedor de contenido), Plaza-Pineda, M. (Proveedor de contenido).	2
Urrutia, N.S. (2019). Caracterización de la mastofauna asociada a los bosques secos del Dagua - Valle del Cauca, 2018. v1.0. Instituto de Investigaciones Ambientales del Pacifico John Von Neumann (IIAP). Dataset/Occurrence.	5
Velazco, P. M., Autino, A. G., &Claps, G. L. (2014). New record of the ectoparasite insect *Speiseria ambigua* Kessel, 1925 (Diptera: Streblidae) of *Platalina genovensium* Thomas, 1928 (Chiroptera: Phyllostomidae) from Peru. Check List, 10(6), 1525–1527.	1
Villegas M., Lozano Zambrano F.H. (2018). Monitoreo y seguimiento de fauna terrestre para el AID y AII de la Central Hidroeléctrica de Calima. 2017-B. v1.1. Corporación Paisajes Rurales. Dataset/Occurrence.	2
Voigt, C. C., and Kelm, D. H. (2006). Host preference of the common vampire bat (*Desmodus rotundus*; Chiroptera) assessed by stable isotopes. Journal of Mammalogy, 87(1), 1–6.	1
Willig, M. R., Presley, S. J., Owen, R. D., &Lopez-González, C. (2000). Composition and structure of bat assemblages in Paraguay: A subtropical temperate interface. Journal of Mammalogy, 81(2), 386–401.	8
Wohlgenant, T. J. (1994). Roost interactions between the common vampire bat (*Desmodus rotundus*) and two frugivorous bats (*Phyllostomus discolor* and *Sturnira lilium*) in Guanacaste, Costa Rica. Biotropica, 26(3), 344–348.	1
Wray, A.K., Olival, K.J., Moran, D., Lopez, M.R., Alvarez, D., Navarrete-Macias, I., Liang, E., Simmons, N.B., Lipkin, W.I., Daszak, P., Anthony, S.J. (2016). Viral diversity, prey preference, and bartonella prevalence in *Desmodus rotundus* in Guatemala. Ecohealth, 13(4), 761–774.	5
Zarza, H., Martinez-Meyer, E., Suzan, G., Ceballos, G. 2017. Geographic distribution of *Desmodus rotundus* in Mexico under current and future climate change scenarios: Implications for bovine paralytic rabies infection. Veterinaria Mexico, 4, 3–16.	1061

The following table lists (alphabetically) the literary and published sources of *Desmodus rotundus* occurrence reports not from publicly available data reservoirs. The references are verbatim (as initially listed by the source) and therefore may not all follow the same citation format. Note that number of occurrences is based on location, not the number of individuals reported at each location if more than one individual was reported.

**Online-only Table 3:** Individual contributors.

Source	Number of Occurrences in Final Dataset
Asociacion de Becarios del Casanare	54
Asociacion para el estudio y conservacion de las aves acuaticas en Colombia - Calidris	2
Colección Mamíferos Lillo (CML), Universidad Nacional de Tucumán, Argentina	173
Colegio de La Frontera Sur (ECOSUR) in Mexico and the University of San Carlos in Guatemala	41
Colegio de La Frontera Sur (ECOSUR) of Mexico	37
Corporacion Paisajes Rurales	4
CRC - Corporacion Autonoma Regional del Valle del Cauca	2
Daniel G. Streicker. Institute of Biodiversity, Animal Health and Comparative Medicine, College of Medical Veterinary and Life Sciences, University of Glasgow, UK.	55
El Servicio Nacional de Sanidad, Inocuidad y Calidad Agroalimentaria (SENASICA)	929
Elsa Cárdenas-Canales. Department of Pathobiological Sciences, School of Veterinary Medicine, University of Wisconsin-Madison, USA	8
Fundacion Chimbilako	3
Fundacion Humedales	1
Fundacion Omacha	3
INERCO Consultora Colombia	1
Instituto Amazonico de Investigaciones Cientãficas - SINCHI	18
Instituto de Investigacion de Recursos Biolãgicos Alexander von Humboldt	191
Instituto de Investigaciones Ambientales del Pacifico John Von Neumann - IIAP	5
Isagen S.A.S. Institute of Colombia	2
Museo Argentino de Ciencias Naturales (MACN) “Bernardino Rivadavia” de Buenos Aires.	85
Museo La Salle (MLS) Bogotá, Colombia.	38
Museo Provincial de Ciencias Naturales “Florentino Ameghino” (MFA-ZV-M), Santa Fe, La Capital	3
Museo Provincial de Ciencias Naturales (MG-ZV-M), Rosario, Santa Fe	1
Natural History Museum, Universidad Nacional Mayor de San Marcos, Peru	467
Naturalista Colombia	4
Parques Nacionales Naturales de Colombia	10
Patrimonio Natural Fondo para la Biodiversidad y Areas Protegidas “Patrimonio Natural”	48
Programa para la Conservación de los Murciélagos de Chile (PCMCh)	334
Ruben Barquez and Monica Diaz. Programa de Investigaciones de Biodiversidad Argentina. Facultad de Ciencias Naturales e Instituto Miguel Lillo. Universidad Nacional de Tucumán. Consejo Nacional de Investigaciones Científicas y Técnicas. Tucumán, Argentina	213
Universidad de Antioquia	3
Universidad de Caldas	31
Universidad de la Amazonia	2
Universidad de Pamplona	1
Universidad del Valle	3
Universidad Industrial de Santander	11
Universidad Nacional de Colombia	171
Universidad Tecnologica del Choco	29
Universidade Federal de Uberlândia	133

Individuals and institutions that contributed to the dataset and the number of occurrence reports included in the final dataset.
