# Supplementary material for: A database of common vampire bat reports
Source: Sci Data. 2022 Feb 16;9:57. doi: 10.1038/s41597-022-01140-9 (PMC8850563; doi:10.1038/s41597-022-01140-9)
Supplement: Supplementary file 1 — Supplementary Materials [file 41597_2022_1140_MOESM1_ESM.docx]

**Supplementary Materials**

**Supplementary Online-only Table 1: Originating datasets contributing to final dataset.** The following datasets or sources contributed one or more occurrences to the download of publicly available occurrences. The acronyms and titles listed under Data Name are the official distinguishers denoted by the original dataset themselves.

| **Source Name** | **Number of Occurrences in Final Dataset** |
| --- | --- |
| [Actualización de la base de datos de la Colección de mamíferos del Museo de Zoología 'Alfonso L. Herrera'](https://www.gbif.org/dataset/5381c29f-b090-488a-8693-0038708c904f) | 119 |
| [Actualización de la base de datos de la Colección Regional Durango (Mammalia)](https://www.gbif.org/dataset/62d15ada-100a-465f-b17b-bb5c8436470a) | 33 |
| [Actualización de la base de datos de la Colección Regional Durango (Mammalia) (Tejido)](https://www.gbif.org/dataset/2388291e-d210-40f5-be53-5b87ac0806c0) | 12 |
| [Actualización de la base de datos del Atlas Mastozoológico de México](https://www.gbif.org/dataset/80d3003c-f762-11e1-a439-00145eb45e9a) | 130 |
| [Actualización de la base de datos del estado de Morelos de la Colección Nacional de Mamíferos del Instituto de Biología, UNAM](https://www.gbif.org/dataset/87213c27-8362-48ee-af75-d636d44eb2f3) | 791 |
| [Actualización y enriquecimiento de las bases de datos del proyecto de evaluación y análisis geográfico de la diversidad faunística de Chiapas](https://www.gbif.org/dataset/805bbcde-f762-11e1-a439-00145eb45e9a) | 280 |
| American Museum of Natural History ([AMNH) Mammal Collections](https://www.gbif.org/dataset/96ca66b4-f762-11e1-a439-00145eb45e9a) | 40 |
| [Análisis de integridad biológica en el Parque Natural Regional (PNR) El Vínculo](https://www.gbif.org/dataset/3dc8c286-9e33-4665-8641-9858af6ba75b) | 4 |
| [Análisis de Integridad Biológica para el Distrito Regional de Manejo Integrado RUT Nativos.Convenio CVC- Univalle 108 de 2017](https://www.gbif.org/dataset/f66b4f25-909e-4b1e-a030-f7f2dba764ae) | 2 |
| [Análisis de Integridad Biológica para el Parque Natural Regional Páramo del Duende. Convenio CVC- Univalle 108 de 2017](https://www.gbif.org/dataset/2d52d641-da1b-4aeb-844b-32ea3322067e) | 1 |
| [Análisis de la heterogeneidad ambiental y conectividad de las áreas naturales del sur del Valle de México](https://www.gbif.org/dataset/7fdb69da-f762-11e1-a439-00145eb45e9a) | 1 |
| [Análisis de las relaciones entre las diversidades alfa, beta y gamma a distintos niveles de escala espacial: Procesos históricos y ecológicos que intervienen. V Etapa](https://www.gbif.org/dataset/8052bdc8-f762-11e1-a439-00145eb45e9a) | 18 |
| [Anfibios, reptiles y mamíferos del corredor biológico del norte de Yucatán depositados en las colecciones de la Escuela Nacional de Ciencias Biológicas](https://www.gbif.org/dataset/7ff8361e-f762-11e1-a439-00145eb45e9a) | 12 |
| [Angelo State Natural History Collections (ASNHC) Mammal specimens (Arctos)](https://www.gbif.org/dataset/2aa02cf8-c402-412c-9ad1-585e1e185bef) | 22 |
| [Avistajes de especies de valor especial en áreas protegidas del noreste de Argentina](https://www.gbif.org/dataset/a6058f47-15ae-412e-b316-912dd6e2ec9b) | 19 |
| [Base de datos de mamíferos de México depositados en colecciones de Estados Unidos y Canadá](https://www.gbif.org/dataset/2dad0cd2-e880-4ec3-90e5-d3f479528cbd) | 172 |
| [Bat species richness in the region of the Central Valleys of Oaxaca, Mexico](https://www.gbif.org/dataset/127d349d-c641-4897-8fe7-4bea2f540709) | 64 |
| [Biodiversidad asociada a la mina de explotación y producción de Ferroníquel Cerro Matoso S.A.](https://www.gbif.org/dataset/72fa796a-29c3-4805-86c3-9380be2cc5a9) | 1 |
| [Biodiversidad asociada al gasoducto Loop San Mateo-Mamonal](https://www.gbif.org/dataset/9a25dd5f-eb12-47f3-8a7e-51b8e3824600) | 20 |
| [Biodiversidad asociada al proyecto de aprovechamiento de recursos minerales Queresas y Porvenir en el municipio de Planeta Rica](https://www.gbif.org/dataset/1ca5dd25-ba19-4e22-9890-2755c51ecc4b) | 6 |
| [Biodiversidad de los mamíferos en el Estado de Michoacán](https://www.gbif.org/dataset/82d3b0c5-d3f4-46af-b54f-a0bbf1980dd2) | 131 |
| [Biodiversidad mastozoológica del Eje Volcánico Transversal](https://www.gbif.org/dataset/1a2b7ede-2c91-4952-afea-dfbf5cd3c677) | 92 |
| [Biodiversity Research and Teaching Collections - TCWC Vertebrates](https://www.gbif.org/dataset/b6015b60-6f96-43a9-88e5-2f41854e8f07) | 2 |
| [Biología del murciélago mastín enano (*Eumops bonariensis* nanus) en Yucatán](https://www.gbif.org/dataset/2b568c96-1792-4c02-8709-569f520ad654) | 3 |
| [Bosque Mesófilo de Montaña de México](https://www.gbif.org/dataset/a5be4674-3ee0-4aab-9ce6-c602a7d71d18) | 14 |
| [California Academy of Science (CAS) Mammalogy (MAM)](https://www.gbif.org/dataset/6ce7290f-47f6-4046-8356-371f5b6749df) | 16 |
| [Canadian Museum of Nature Mammal Collection](https://www.gbif.org/dataset/f86a681d-7db8-483b-819a-248def18b70a) | 2 |
| [Caracterización biológica de la ventana de biodiversidad Ciénaga, Municipio de Ciénaga, Magdalena, Colombia](https://www.gbif.org/dataset/8db77d0a-f139-4f1c-b1c3-16c7fd4a17c0) | 1 |
| [Caracterización biológica de la ventana de biodiversidad del Complejo Cenagoso de Zapatosa, Municipio de Chimichagua, Cesar, Colombia](https://www.gbif.org/dataset/689385b6-a672-4919-a4cc-3365c9d5aceb) | 1 |
| [Caracterización biológica de la ventana de biodiversidad enclave Atuncela, municipio de Dagua, Valle del Cauca](https://www.gbif.org/dataset/d60abc0b-d936-40eb-a696-d54b9499e493) | 4 |
| [Caracterización biológica de la ventana de biodiversidad Montes de María, Municipio de Colosó, Sucre, Colombia](https://www.gbif.org/dataset/4df57e87-369b-418b-b9a4-5b69fc64857c) | 9 |
| [Caracterización biológica de la ventana de biodiversidad municipio de Filandia, Quindío, Colombia](https://www.gbif.org/dataset/d52b82ef-d3b9-47e3-802b-5725659812a6) | 3 |
| [Caracterización biológica del Monumento Natural Yaxchilán como un elemento fundamental para el diseño de su plan rector de manejo](https://www.gbif.org/dataset/5dbf40d0-0d2d-46af-a205-c83ff2e74098) | 1 |
| [Caracterización biológica en la Serranía de San Lucas 2015](https://www.gbif.org/dataset/bdb3951b-1b8a-446d-8820-22ce0a2ea380) | 2 |
| [Caracterización biótica para la conservación de especies amenazadas en el área de influencia del Oleoducto Bicentenario, departamentos de Arauca y Casanare, Colombia](https://www.gbif.org/dataset/b90cadd7-9ef5-4640-b1fe-94d4803e7129) | 2 |
| [Caracterización de Fauna del Banco de Hábitat del Bosque Seco Tropical Antioquia de Terrasos](https://www.gbif.org/dataset/a9255982-bbf4-4185-808a-30ad4ea4aab9) | 7 |
| [Caracterización de fauna para el establecimiento de nuevas Reservas Naturales de la Sociedad Civil en Arauca](https://www.gbif.org/dataset/1ef66bdc-801a-4e64-b83a-66125d1aebab) | 5 |
| [Caracterización de fauna para la formulación y actualización de Planes de Manejo Ambiental en la jurisdicción de Corpoguavio](https://www.gbif.org/dataset/5355d3bf-9225-455b-9a54-5b2524b15287) | 1 |
| [Caracterización de Fauna y Flora en ecosistemas de piedemonte, montaña y humedales en el departamento del Casanare](https://www.gbif.org/dataset/e550b7a5-439c-42d6-917a-5990a633347d) | 1 |
| [Caracterización de fauna y flora para el establecimiento de límites funcionales de humedales en tres ventanas piloto: Ciénaga de la Virgen, Ciénaga Zapatosa y Complejo de humedales Paz de Ariporo - Hato Corozal](https://www.gbif.org/dataset/8bc4f5d2-8e7a-430d-b219-6776c38f401a) | 1 |
| [Caracterización de Flora y Fauna en la cuenca del río Calamar, municipios de Bolívar y Trujillo, Valle del Cauca](https://www.gbif.org/dataset/e61f1cc7-d7cd-4884-a4b8-b41de46d101c) | 2 |
| [Caracterización de la mastofauna asociada a los bosques secos del Dagua - Valle del Cauca, 2018](https://www.gbif.org/dataset/3e8cc719-e0e9-45ec-8341-4b80ae0e6eb3) | 5 |
| [Caracterización faunística de la Reserva Forestal Protectora Regional Brisas del Rincón](https://www.gbif.org/dataset/2fdace5f-efaa-4f5c-8680-acb8f7797319) | 1 |
| Caribbean Public Health Agency ([CAREC) Mammals](https://www.gbif.org/dataset/30a29818-d6d2-4116-b59b-1facb9276526) | 2 |
| [Catálogo de los mamíferos de México en resguardo de The Natural History Museum (London), Inglaterra](https://www.gbif.org/dataset/e9ba8fcf-8aac-4c98-a415-a25e9bacd54c) | 27 |
| [CNMA/Colección de Mamíferos de la Estación de Biología Chamela](https://www.gbif.org/dataset/96b422d2-f762-11e1-a439-00145eb45e9a) | 4 |
| [CNMA/Colección de Mamíferos de la Estación de Biología Los Tuxtlas](https://www.gbif.org/dataset/96b2e606-f762-11e1-a439-00145eb45e9a) | 10 |
| [CNMA/Colección Nacional de mamíferos](https://www.gbif.org/dataset/84faa3a4-f762-11e1-a439-00145eb45e9a) | 989 |
| [Coleção de Mamíferos da Universidade Estadual do Norte Fluminense](https://www.gbif.org/dataset/20e7ce8d-ec4f-4de8-a6b1-c16134d0a5bf) | 8 |
| [Coleção Zoológica de Referência da Universidade Federal de Mato Grosso do Sul - Chiroptera (ZUFMS-CHI)](https://www.gbif.org/dataset/4d73eb02-92ec-41a2-898e-acf8344cee2e) | 6 |
| [Colección de Mamíferos – Universidad de la Amazonia](https://www.gbif.org/dataset/8ffeca3a-6ef4-43fb-8319-d82fe17dccc7) | 2 |
| [Colección de Mamíferos - Universidad del Quindío](https://www.gbif.org/dataset/f4202f2a-e5bf-44a6-a0f1-cc0f3f3f4f3e) | 2 |
| [Colección de Mamíferos (Mammalia) del Museo de Historia Natural de la Universidad de Caldas, Colombia](https://www.gbif.org/dataset/1a58ec89-e5d4-4b2b-9ed3-1c8b5f70f4fc) | 27 |
| [Colección de mamíferos de la Universidad del Valle](https://www.gbif.org/dataset/64a88778-8de7-4dd0-af39-2d094102cbde) | 28 |
| [Colección de Mamíferos del Instituto de Ciencias Naturales (ICN-MHN-Ma)](https://www.gbif.org/dataset/3d88255b-67ae-4f82-899b-06186eab2995) | 100 |
| [Colección de Mamíferos del Instituto de Investigación de Recursos Biológicos Alexander von Humboldt (IAvH-M)](https://www.gbif.org/dataset/2a337d5a-a784-4f8a-a947-46d69e0fa3eb) | 17 |
| [Colección de Mamíferos del Museo de Historia Natural C.J. Marinkelle](https://www.gbif.org/dataset/4cb6c022-7654-4901-bb3d-e7028a26b1ac) | 2 |
| [Colección de mamíferos del Museo de Historia Natural de la Pontificia Universidad Javeriana](https://www.gbif.org/dataset/4dd6694a-2b15-464e-ae46-3cfb683e821e) | 35 |
| [Colección de Tejidos del Instituto de Investigación de Recursos Biológicos Alexander von Humboldt (IAvH-CT)](https://www.gbif.org/dataset/9c2d26f1-0ce0-4cde-9f8d-de8284404fff) | 34 |
| [Colección de Zoología General de la Universidad de Pamplona](https://www.gbif.org/dataset/2196fd54-22a3-406e-8480-cbee25349024) | 1 |
| [Colección Mastozoológica de la Universidad Distrital Francisco José de Caldas](https://www.gbif.org/dataset/7f9161a5-3d90-4d07-9e95-433f0d9e0afe) | 22 |
| [Colección Mastozoológica de la Universidad Industrial de Santander](https://www.gbif.org/dataset/83964202-07d9-4a27-8391-975144bcc1da) | 11 |
| [Colección Mastozoológica del Museo de Historia Natural de la Universidad del Cauca](https://www.gbif.org/dataset/bbaad610-29b7-480e-a7bc-f5a7dc10c191) | 3 |
| [Colección Mastozoológica Universidad de los Llanos](https://www.gbif.org/dataset/efedb425-09ae-4220-831b-51f7b1ab48eb) | 8 |
| [Colección Quirópteros Museo de La Salle Bogotá (MLS)](https://www.gbif.org/dataset/dc68ef79-97f5-4984-9dc4-c463c7be5870) | 10 |
| [Colección Teriológica de la Universidad Tecnológica del Chocó](https://www.gbif.org/dataset/953c792e-1417-4d0a-97b6-b0a1d7c96f0d) | 26 |
| [Colección Teriológica Universidad de Antioquia](https://www.gbif.org/dataset/8dc1f1ac-8d4f-4d5b-bbde-a5028143f37f) | 3 |
| [Colección Zoológica de la Universidad del Tolima (CZUT)- Mastozoología](https://www.gbif.org/dataset/87c22676-55df-4e72-b2fa-6488700576d1) | 48 |
| Colegio de La Frontera Sur (ECOSUR) in Mexico and the University of San Carlos in Guatemala. | 4 |
| Colegio de La Frontera Sur (ECOSUR) of Mexico. | 37 |
| [Computarización de la colección de mamíferos del Centro de Educación Ambiental e Investigación Sierra de Huautla (CEAMISH) de la Universidad Autónoma del Estado de Morelos (UAEM)](https://www.gbif.org/dataset/f0d7eb48-a312-4643-a1bb-1ba9370497ec) | 1 |
| [Computarización de la Colección de mamíferos del Centro de Investigaciones Biológicas del Noroeste SC](https://www.gbif.org/dataset/3167cfa0-50fa-4037-bc92-1791fabb4694) | 4 |
| [Computarización de las colecciones de vertebrados terrestres de la Escuela Nacional de Ciencias Biológicas, IPN - Fases 2](https://www.gbif.org/dataset/801c8370-f762-11e1-a439-00145eb45e9a) | 16 |
| [Computarización de las colecciones de vertebrados terrestres de la Escuela Nacional de Ciencias Biológicas, IPN - Fases 3](https://www.gbif.org/dataset/7fe6b042-f762-11e1-a439-00145eb45e9a) | 64 |
| [Computarización de las colecciones de vertebrados terrestres de la Escuela Nacional de Ciencias Biológicas, IPN Fase 4 (Mamíferos)](https://www.gbif.org/dataset/804a818f-c1e8-4347-a2f2-02a5f22e1a82) | 510 |
| Cornell University Museum of Vertebrates ([CUMV) Mammal Collection](https://www.gbif.org/dataset/35720b3e-aded-4b83-b4f1-967f1d457d6a) | 2 |
| [Deforestación y fragmentación del hábitat: consecuencias ecológicas sobre la fauna de mamíferos de la selva tropical estacional](https://www.gbif.org/dataset/7fffa0a2-f762-11e1-a439-00145eb45e9a) | 1 |
| [Diagnóstico y monitoreo de la diversidad biológica de las sabanas inundables en Casanare. Primera etapa: mamíferos y aves](https://www.gbif.org/dataset/3e85137e-ac2d-4dac-8531-f54ab62b01a2) | 20 |
| [Distribución geográfica de las aves y los mamíferos de las zonas montanas de los estados de San Luis Potosí e Hidalgo circundantes de la Sierra Gorda](https://www.gbif.org/dataset/7fabfa2e-f762-11e1-a439-00145eb45e9a) | 3 |
| [Distribución geográfica de las aves y los mamíferos del estado de Querétaro](https://www.gbif.org/dataset/7fa49f72-f762-11e1-a439-00145eb45e9a) | 97 |
| [Diversidad de grupos selectos de flora (Magnoliopsida: Malvales, Sapindales, Fabales, Laurales, Solanales y Caryophyllales) y fauna (Lepidoptera, Diptera, Odonata, Amphibia, Reptilia, Aves y Mammalia) del APFF Boquerón de Tonalá, Oaxaca, México](https://www.gbif.org/dataset/b14cd984-dfc4-499d-b1bd-650ceef3608a) | 2 |
| [Diversidad de los mamíferos de la Reserva de la biósfera Tehuacán-Cuicatlán, Puebla-Oaxaca, México](https://www.gbif.org/dataset/7fd54c8a-f762-11e1-a439-00145eb45e9a) | 33 |
| [Diversidad y distribución mastofaunística en las regiones prioritarias para la conservación del estado de Oaxaca](https://www.gbif.org/dataset/d0d7ddf4-c5c9-4049-b6a5-9130eadc014a) | 119 |
| [DMNS Mammal Collection (Arctos)](https://www.gbif.org/dataset/5f3463d2-51b6-4a8a-b252-9bab4388934e) | 1 |
| [Données d'occurrences Espèces issues de l'inventaire des ZNIEFF](https://www.gbif.org/dataset/f946666e-67dc-4848-9fa8-2162f3559e33) | 11 |
| [Données naturalistes de Frédéric LEBLANC](https://www.gbif.org/dataset/4c6575de-cac0-4bce-a71f-e7f73984717a) | 17 |
| [Establecimiento de un área protegida en el Alto y Bajo Calima, área clave de biodiversidad en el Valle del Cauca, Colombia](https://www.gbif.org/dataset/d4ba9607-189b-4c35-af5c-0b6ca3a54d50) | 1 |
| [Estación Biológica de Doñana - CSIC, Mammal Collection](https://www.gbif.org/dataset/85facc5c-f762-11e1-a439-00145eb45e9a) | 44 |
| [Estructura Ecológica de la Subregión Norte de Caldas, Contrato para actividades científicas y tecnológicas No. 222-2018 entre Corpocaldas y WCS](https://www.gbif.org/dataset/71a0da97-201f-4fee-982a-d138e21a0ecb) | 5 |
| [Estudio de Biodiversidad Mina Calenturitas, Temporada de Lluvias](https://www.gbif.org/dataset/db191f3a-48ed-4b94-93ef-5c0517fb1b58) | 1 |
| [Fauna asociada (endocárstica) a cuevas en el municipio de El Peñón, Santander - Proyecto Colombia BIO](https://www.gbif.org/dataset/8aad9e02-2337-4836-83d6-bb505b2b2d11) | 8 |
| [Fauna y flora asociada a 18 humedales del valle geográfico del río Cauca](https://www.gbif.org/dataset/969597cd-473c-4d18-90e8-d11b5c107d0a) | 2 |
| [Fauna y Flora de Cinaruco - 2014- 2016](https://www.gbif.org/dataset/2bee4a91-9d92-430e-a87e-ab32d3b1ce47) | 10 |
| [Fauna y flora de la cuenca media del río Lebrija en Rionegro, Santander](https://www.gbif.org/dataset/1cd2fdd6-24d3-4402-905f-8a72ddffd3ad) | 1 |
| [Fauna y flora en áreas con cultivos de palma de aceite en el norte y oriente de Colombia](https://www.gbif.org/dataset/09d5405e-ca86-45f2-afa7-93cc707f54aa) | 9 |
| [Field Museum of Natural History (Zoology) Mammal Collection](https://www.gbif.org/dataset/41fc5c40-5e81-496f-9733-6b5681b3b7a5) | 874 |
| Fonoteca Neotropical Jacques Vielliard ([FNJV)](https://www.gbif.org/dataset/ca2e422f-be48-42a5-bca5-a37cada435c8) | 1 |
| [Formación de las colecciones de referencia de aves y mamíferos de la Reserva de la Biosfera de Sian Ka'an, Quintana Roo, México](https://www.gbif.org/dataset/dd83b173-0b9f-4262-a49d-c72982965e69) | 2 |
| Fort Hayes Sternberg Museum of Natural History ([FHSM) Mammals Collection](https://www.gbif.org/dataset/84e6e5f8-f762-11e1-a439-00145eb45e9a) | 42 |
| [Fortalecimiento de las colecciones de ECOSUR. Primera fase (Mamíferos San Cristóbal)](https://www.gbif.org/dataset/cb1a521c-29f7-4751-8661-898d16d0caa6) | 53 |
| [Geographically tagged INSDC sequences](https://www.gbif.org/dataset/ad43e954-dd79-4986-ae34-9ccdbd8bf568) | 142 |
| [Historia natural del parque ecológico estatal de Omiltemi, Chilpancingo, Guerrero, México](https://www.gbif.org/dataset/8014fede-f762-11e1-a439-00145eb45e9a) | 3 |
| [ICN - Universidad Nacional de Colombia](https://www.gbif.org/dataset/79684ec1-01e8-46cc-83cb-cd5bdfb469fe) | 60 |
| [iNaturalist Research-grade Observations](https://www.gbif.org/dataset/50c9509d-22c7-4a22-a47d-8c48425ef4a7) | 181 |
| [Incorporación de nuevos registros de mamíferos de la región Sierra Norte de Oaxaca](https://www.gbif.org/dataset/80a24726-f762-11e1-a439-00145eb45e9a) | 19 |
| [International Barcode of Life project (iBOL)](https://www.gbif.org/dataset/040c5662-da76-4782-a48e-cdea1892d14c) | 127 |
| [Inventario de Aves y Mamíferos en humedales del departamento de Córdoba](https://www.gbif.org/dataset/a1a94e37-171d-41b0-a65d-b75664d4f9c0) | 1 |
| [Inventario de Fauna Vertebrada de la Reserva Forestal Protectora Regional de los Cañones de los Ríos Melcocho y Santo Domingo](https://www.gbif.org/dataset/d628bc9c-af63-49af-91b3-1a63c603feb2) | 1 |
| [Inventario de los mamíferos de las reservas de la biósfera Mapimí, La Michilía, El Cielo y Calakmul](https://www.gbif.org/dataset/807e6f90-f762-11e1-a439-00145eb45e9a) | 11 |
| [Inventario de los murciélagos de las regiones áridas y semiáridas de México y la elaboración de su catálogo de sonogramas](https://www.gbif.org/dataset/5bfde5fb-4beb-45c9-922f-f897fe8cc710) | 109 |
| [Inventario multitaxonómico: PN El Potosí y RB Sierra del Abra Tanchipa (San Luis Potosí)](https://www.gbif.org/dataset/7d0775ec-119a-43ca-8121-cdb142869169) | 18 |
| [Inventario y evaluación de fauna silvestre en el campo de exploración petrolera Niscota Sur](https://www.gbif.org/dataset/75d2949e-e924-4af5-9b1b-0fd5172e32a8) | 2 |
| [Inventarios de Fauna y Flora en Relictos de Bosque en el Enclave Seco del Río Amaime, Valle del Cauca](https://www.gbif.org/dataset/fdbbb939-ee0b-4c6e-8eb3-15ed92177306) | 6 |
| [Inventarios de flora y fauna en el piedemonte de los municipios Aguazul, Tauramena y Yopal del departamento de Casanare](https://www.gbif.org/dataset/f1caac33-15ac-4185-90df-aec093abc0cd) | 9 |
| [Inventarios de la biodiversidad en el piedemonte subandino de Yopal](https://www.gbif.org/dataset/51d648b8-5d7a-4233-b491-d00a8d54b215) | 2 |
| [Inventarios de Murciélagos de la Microcuenca de la Quebrada Agua Blanca, Vereda Marroquín, Yopal, Casanare](https://www.gbif.org/dataset/f0090be2-6b7f-40aa-9963-b8a1f749aa72) | 6 |
| [La mastofauna del cuaternario tardío de México](https://www.gbif.org/dataset/987fc6a5-1a11-452b-be14-b89e027fa3e4) | 19 |
| [Línea base biótica del proyecto de Restauración Ecológica Re-Viva La Primavera](https://www.gbif.org/dataset/6fea96d6-e47f-4cd5-abcd-08235a8e26d7) | 9 |
| [Línea base del medio biótico en los bosques dentro de la zona propuesta para Acuerdos de Conservación Voluntaria en la microcuenca de la Quebrada Aguazula - Rincón del Soldado_Yopal](https://www.gbif.org/dataset/b2334c74-b73f-4cfb-b088-683119fa3c6e) | 3 |
| [Línea base del medio biótico en los bosques para Acuerdos de Conservación Voluntaria en la microcuenca de la Quebrada Aguablanca -El Morro Yopal](https://www.gbif.org/dataset/dd223a19-c7f6-4847-9422-614c6e8bc623) | 6 |
| Los Angeles County Museum of Natural History ([LACM) Vertebrate Collection](https://www.gbif.org/dataset/7a25f7aa-03fb-4322-aaeb-66719e1a9527) | 509 |
| Louisiana Museum of Natural History ([LSUMZ) Mammals Collection](https://www.gbif.org/dataset/847e2306-f762-11e1-a439-00145eb45e9a) | 172 |
| [Mamíferos asociados a cuatro ventanas de la Orinoquia Colombiana - SULU I](https://www.gbif.org/dataset/cd77bcbf-a35d-4e6a-a361-fd5634295c20) | 1 |
| [Mamíferos colectados en la transición Andino-Amazónica del departamento del Caquetá - Proyecto Colombia BIO](https://www.gbif.org/dataset/2ca1c052-4c80-4eb6-96b8-e71b9f761b36) | 1 |
| [Mamíferos de 14 municipios del Departamento del Valle del Cauca para el fortalecimiento de RNSC y Caracterización de Predios Adquiridos para la Conservación de Cuencas](https://www.gbif.org/dataset/3546c1aa-a3e2-4888-870f-b246cd0971ed) | 20 |
| [Mamíferos de los Parques Nacionales Lagunas de Montebello y Palenque, Chiapas](https://www.gbif.org/dataset/8088b072-f762-11e1-a439-00145eb45e9a) | 161 |
| [Mamíferos de Nuevo León: distribución y taxonomía](https://www.gbif.org/dataset/91c888e7-3fac-4d2b-9c24-3b3d2687bfa5) | 91 |
| [Mamíferos del Estado de Veracruz](https://www.gbif.org/dataset/bbee2ef2-1389-43e6-bde8-ac8e38c6cc6e) | 110 |
| [Mamíferos del piedemonte de la sabana de la Orinoquia](https://www.gbif.org/dataset/90485473-b608-42f3-bf21-15b34c46c24d) | 10 |
| [Mamíferos laguna El Tinije](https://www.gbif.org/dataset/342dd753-d406-499e-8f7a-41c1e228276d) | 1 |
| [Mamíferos pequeños en el municipio de Medina, Cundinamarca - Proyecto Colombia Bio](https://www.gbif.org/dataset/f8b0b52b-0383-44dd-9940-851e80bf6131) | 10 |
| [Mamíferos silvestres de la cuenca del río Mezquital-San Pedro, Durango-Nayarit](https://www.gbif.org/dataset/f75482a0-f244-4bd2-aff9-92130e99bbe2) | 14 |
| [Mamíferos voladores laguna El Tinije](https://www.gbif.org/dataset/acdf883a-82fc-4181-bbdc-6f47d46e8b6c) | 248 |
| [Mammalogy Collection - Royal Ontario Museum](https://www.gbif.org/dataset/c5c4a23e-2035-4416-ab64-032d6df52ddb) | 835 |
| [Mammals housed at MHNG, Geneva](https://www.gbif.org/dataset/5a659248-1f70-11e3-b2c5-00145eb45e9a) | 118 |
| [MBML-Mamiferos - Coleção de Mamíferos](https://www.gbif.org/dataset/2177d641-eeca-41c0-b6ab-0f1b2dcd9a79) | 245 |
| [Monitoreo Ambiental del Proyecto La Colosa](https://www.gbif.org/dataset/9cc67633-537a-4acc-8d37-92feeaddb2cd) | 4 |
| [Monitoreo de Flora y Fauna de los Acuerdos de Conservación Voluntaria como estrategia de inversión del 1% de Equión Energía Limited](https://www.gbif.org/dataset/73416d1c-e675-43b3-bbe4-672d5a9f9e8b) | 16 |
| [Monitoreo de la Fauna Silvestre con Capacitación y Certificación de Monitores Comunitarios en las Microrregiones Prioritarias para la Conectividad y Conservación de la Biodiversidad Agua Blanca, Cañón del Usumacinta, Sierra de Tabasco](https://www.gbif.org/dataset/f4b27145-384c-4c5a-8d28-af9310a37412) | 3 |
| [Monitoreo y seguimiento de fauna terrestre para el AID y AII de la Central Hidroeléctrica de Calima. 2017-B.](https://www.gbif.org/dataset/da285c2a-0cf0-4637-87d8-6e2b5cd8ea01) | 4 |
| Moore Laboratory of Zoology ([MLZ) Mammal Collection (Arctos)](https://www.gbif.org/dataset/226b536c-25f9-4f6b-b144-edcbdffa3566) | 15 |
| [MSU Mammalogy, Ornithology and Vertebrate Paleontology Collections](https://www.gbif.org/dataset/22a66350-7947-4a49-84a3-39c7c1b0881f) | 100 |
| [Murciélagos de las sabanas inundables de las cuencas de los ríos Bita, Manacacías y Cravo Sur - SULU II](https://www.gbif.org/dataset/d842fe12-004c-4081-8620-c1de000b1e28) | 2 |
| [Murciélagos observados en la transición Andino Amazónica del departamento del Caquetá - Proyecto Colombia BIO](https://www.gbif.org/dataset/086abfe4-f8c7-439c-a462-3f3453fa0a11) | 17 |
| [Museo Argentino de Ciencias Naturales "Bernardino Rivadavia" (MACN). Mammalogy National Collection (MACNMa)](https://www.gbif.org/dataset/512c11b0-a103-11de-88ae-b8a03c50a862) | 89 |
| Museo La Salle (MLS) Bogotá, Colombia. | 38 |
| [Museum of Comparative Zoology, Harvard University](https://www.gbif.org/dataset/4bfac3ea-8763-4f4b-a71a-76a6f5f243d3) | 96 |
| Museum of Southwestern Biology ([MSB) Mammal Collection (Arctos)](https://www.gbif.org/dataset/b15d4952-7d20-46f1-8a3e-556a512b04c5) | 134 |
| Museum of Vertebrate Zoology ([MVZ) Mammal Collection (Arctos)](https://www.gbif.org/dataset/0daed095-478a-4af6-abf5-18acb790fbb2) | 221 |
| National Museum of Natural History ([NMNH) Extant Specimen Records](https://www.gbif.org/dataset/821cc27a-e3bb-4bc5-ac34-89ada245069d) | 612 |
| Natural History Museum of Utah ([UMNH) Mammals Collection (Arctos)](https://www.gbif.org/dataset/06a00852-f764-4fb8-80d4-ca51f0918459) | 1 |
| Natural History Museum, Universidad Nacional Mayor de San Marcos, Peru | 467 |
| [Naturgucker](https://www.gbif.org/dataset/6ac3f774-d9fb-4796-b3e9-92bf6c81c084), citizen science nature observations | 2 |
| North Carolina Museum of Natural Sciences ([NCSM) Mammals Collection](https://www.gbif.org/dataset/ea9f5b0b-ad97-45ea-935a-ba2784c80cbb) | 2 |
| [Observation.org, Nature data from around the World](https://www.gbif.org/dataset/8a863029-f435-446a-821e-275f4f641165) | 5 |
| [Oklahoma Collection of Genomic Resources Tissues Specimens](https://www.gbif.org/dataset/71e82020-f762-11e1-a439-00145eb45e9a) | 14 |
| [P. W. Lund collection in the National History Museum of Denmark, Copenhagen](https://www.gbif.org/dataset/84d8287e-f762-11e1-a439-00145eb45e9a) | 1 |
| [Paleobiology Database](https://www.gbif.org/dataset/bb5b30b4-827e-4d5e-a86a-825d65cb6583) | 3 |
| [Patrones de diversidad florística y faunística del área focal Ixcan, selva Lacandona, Chiapas (Mamíferos)](https://www.gbif.org/dataset/7fd7b40c-f762-11e1-a439-00145eb45e9a) | 2 |
| [Peces y Mamíferos de la región de Norogachi, Alta Sierra Tarahumara, Chihuahua (Mamíferos)](https://www.gbif.org/dataset/3679494f-e9d3-4334-9cf7-02726df790ea) | 21 |
| [Proyecto COL88611 para la conservación y uso sostenible de ecosistemas secos](https://www.gbif.org/dataset/31da52cd-6114-439d-be0a-700bf52de8f3) | 48 |
| [Proyecto interno ejecutado por la CONABIO: Sistema de información sobre Bosque Mesófilo de Montaña de México para apoyo en programas de restauración (Fase 1)](https://www.gbif.org/dataset/dd08f438-2915-424a-a861-873eaa031b95) | 3 |
| [Quirópteros del Parque Natural Regional El Vínculo y su zona de amortiguación (Buga, Valle del Cauca, Colombia)](https://www.gbif.org/dataset/96e5a2f0-d008-4136-955c-83cd139b23c6) | 27 |
| [Rapid Assessment Program (RAP) Biodiversity Survey Database](https://www.gbif.org/dataset/4d749d70-e2e1-11dd-8102-b8a03c50a862) | 17 |
| [RBINS DaRWIN](https://www.gbif.org/dataset/8138eb72-f762-11e1-a439-00145eb45e9a) | 1 |
| [Registro de murciélagos del Cañón del Chicamocha, Santander (2013 - 2014)](https://www.gbif.org/dataset/0bd0f5f0-c661-4ed0-a53f-eb3319d83c87) | 3 |
| [Registros biológicos de especies de fauna vertebrada terrestre en las centrales de San Carlos y Jaguas - 2009](https://www.gbif.org/dataset/983c4ccb-377d-412f-9eb8-6d96d0c620fa) | 2 |
| [Registros de quirópteros de tres localidades del caribe colombiano](https://www.gbif.org/dataset/a0b54745-9319-47e2-b137-86c49edbc1a6) | 25 |
| Sam Noble Museum [Mammals Specimens](https://www.gbif.org/dataset/84aefc7e-f762-11e1-a439-00145eb45e9a) | 271 |
| [Sistema de Informação sobre a Biodiversidade Brasileira](https://www.gbif.org/publisher/f5fd374b-89cb-4ab6-b3eb-794c65f232c3) ([fauna_do_municipio_de_araraquara-sp](https://www.gbif.org/dataset/fe7657bc-aa54-4b5d-b084-f5c91275ad62)) | 1 |
| [Sistematización de las colecciones científicas del Instituto de Historia Natural y Ecología, (IHNE) Chiapas](https://www.gbif.org/dataset/800ea6f6-f762-11e1-a439-00145eb45e9a) | 22 |
| Slater Museum of Natural History ([PSM) Vertebrates Collection](https://www.gbif.org/dataset/8eddc200-f535-4c65-9b4d-f723eafe607e) | 2 |
| [Tejidos colectados en la ventana de biodiversidad Ciénaga, Municipio de Ciénaga, Magdalena, Colombia](https://www.gbif.org/dataset/a797f77c-cd90-401c-827c-92662081cd1d) | 1 |
| [Tejidos colectados en la ventana de biodiversidad enclave Atuncela, municipio de Dagua, Valle del Cauca](https://www.gbif.org/dataset/c3155684-157a-49d9-b63d-87fb4b63a361) | 2 |
| [Tejidos colectados en la ventana de biodiversidad Filandia, Quindío, Colombia](https://www.gbif.org/dataset/e6d89684-dc2b-47f6-9ed8-1596b46f3025) | 1 |
| [Tejidos de ejemplares colectados en el Páramo de Chingaza - Proyecto Colombia Bio](https://www.gbif.org/dataset/a8e534dd-7b00-458c-a4c7-ca539ea28ff8) | 10 |
| [Tejidos de quirópteros de tres localidades del caribe colombiano](https://www.gbif.org/dataset/02aeca0a-7e26-416d-b6d6-924807d0112c) | 24 |
| Texas Tech University ([TTU) Mammals Collection](https://www.gbif.org/dataset/854f70cc-55e3-4af2-9417-0f47d6c7902d) | 491 |
| The University of the West Indies ([UWIZM) Mammals](https://www.gbif.org/dataset/24a25949-4531-428d-885d-ae402177440c) | 3 |
| [UCLA Donald R. Dickey Bird and Mammal Collection](https://www.gbif.org/dataset/8631295a-f762-11e1-a439-00145eb45e9a) | 45 |
| [Universidad del Valle de Guatemala - Colecciones Biológicas - Mamíferos](https://www.gbif.org/dataset/3f9f5f30-ab03-49de-8652-f75965133fcd) | 3 |
| [University of Alaska Museum (UAM) Mammal Collection (Arctos)](https://www.gbif.org/dataset/377be098-626f-4cc2-b4b5-35700050669a) | 1 |
| University of Arizona Museum of Natural History ([UAZ) Mammals](https://www.gbif.org/dataset/96275bf2-8999-4869-95d5-4903a84391b0) | 103 |
| University of Connecticut ([UConn) Mammals](https://www.gbif.org/dataset/0b33986b-9b62-451e-ba7a-9c2e777e5580) | 16 |
| [University of Iowa SUI Vertebrate Collection](https://www.gbif.org/dataset/04169fce-7d4f-41f7-9dff-245960f5b59e) | 2 |
| University of Kansas, Biodiversity Institute ([KUBI) Mammalogy Collection](https://www.gbif.org/dataset/1d04e739-98a9-4e16-9970-8f8f3bf9e9e3) | 1160 |
| [University of Michigan Museum of Zoology, Division of Mammals](https://www.gbif.org/dataset/6d2cfc0a-9903-40b8-802b-403398218e4a) | 321 |
| University of Texas at El Paso ([UTEP) Mammals (Arctos)](https://www.gbif.org/dataset/c053c6ad-e590-4c69-a141-5fd9c8ebbe3c) | 3 |
| University of Washington Burke Museum ([UWBM) Mammalogy Collection (Arctos)](https://www.gbif.org/dataset/830eb5d0-f762-11e1-a439-00145eb45e9a) | 28 |
| University of Wyoming Museum of Vertebrates ([UWYMV) Mammal Collection (Arctos)](https://www.gbif.org/dataset/f36b26ac-97d1-40f6-a9a0-484defff98e7) | 2 |
| [USAC (Museo de Historia Natural de la USAC) Mammals Collection](https://www.gbif.org/dataset/fee15ebc-27ce-4aff-b912-2657bbd493d2) | 46 |
| [Vertebrados de los humedales de La Mojana, Colombia](https://www.gbif.org/dataset/7b384b30-7ced-4784-9fc1-1a8e21fa0a01) | 2 |
| [Vertebrados de valor especial en áreas protegidas de la argentina](https://www.gbif.org/dataset/e2483380-16f7-11df-b5b3-b8a03c50a862) | 1 |
| [Vertebrados terrestres de San Juan de Camarones, Durango](https://www.gbif.org/dataset/3a489e55-ec80-4c62-b6aa-4911372d23c2) | 11 |
| [Vertebrados terrestres del Corredor Biológico Sierra Madre del Sur, Chiapas, México](https://www.gbif.org/dataset/802f2cdc-f762-11e1-a439-00145eb45e9a) | 31 |
| [Vertebrados terrestres del parque nacional Cañón del Sumidero, Chiapas, México](https://www.gbif.org/dataset/80425e9c-f762-11e1-a439-00145eb45e9a) | 9 |
| [Vertebrate Zoology Division-Mammalogy, Yale Peabody Museum](https://www.gbif.org/dataset/854f602e-f762-11e1-a439-00145eb45e9a) | 25 |
| [ZUEC-MAM - Coleção de Mamíferos do Museu de Zoologia da UNICAMP](https://www.gbif.org/dataset/8cc8f167-d9eb-4b50-905a-34895bb13325) | 31 |

**
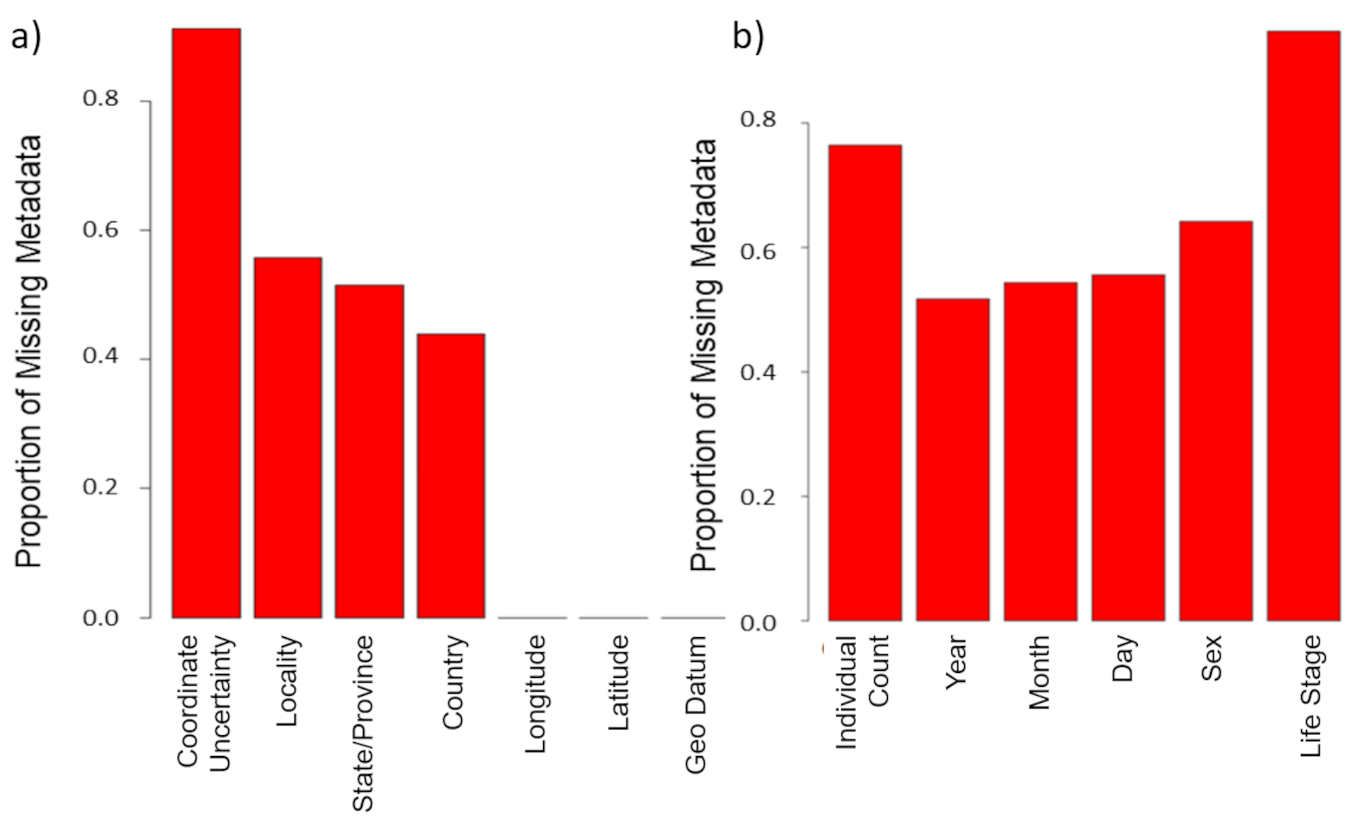
**

**Supplementary Figure 1: Metadata missing from final dataset.** a) Proportion of *Desmodus rotundus* occurrence reports with missing location metadata including uncertainty of the geographic coordinate (Coordinate Uncertainty), location description information (Locality), the administrative state or province of the occurrence report (State/Province), the country where the occurrence report originated (Country), the geographic Longitude of the occurrence report (Longitude), the geographic Latitude of the occurrence report (Latitude), and the geographic spatial reference system used for the occurrence report (Geo Datum). b) Proportion of final *Desmodus rotundus* occurrence reports with missing metadata, including number of individual bats found per site (Individual Count), year of the reported occurrence (Year), month of the reported occurrence (Month), day of the reported occurrence (Day), sex of the individual(s) reported (Sex), and life stage of the reported individual (Life Stage).
